# RAD sequencing of common whelk, *Buccinum undatum,* reveals fine‐scale population structuring in Europe and cryptic speciation within the North Atlantic

**DOI:** 10.1002/ece3.7219

**Published:** 2021-02-09

**Authors:** Jake Goodall, Kristen Marie Westfall, Hildur Magnúsdóttir, Snæbjörn Pálsson, Erla Björk Örnólfsdóttir, Zophonías O. Jónsson

**Affiliations:** ^1^ Faculty of Life and Environmental Sciences University of Iceland Reykjavik Iceland; ^2^ Department of Aquaculture and Fish Biology Hólar University Sauðárkrókur Iceland; ^3^ Vör – Marine Research Center in Breiðafjörður Ólafsvík Iceland; ^4^ Fisheries and Oceans Canada Pacific Biological Station Nanaimo BC Canada

**Keywords:** divergence, *F*_ST_, genetics, phylogeography, speciation

## Abstract

*Buccinum undatum* is a subtidal gastropod that exhibits clear spatial variation in several phenotypic shell traits (color, shape, and thickness) across its North Atlantic distribution. Studies of spatial phenotypic variation exist for the species; however, population genetic studies have thus far relied on a limited set of mitochondrial and microsatellite markers. Here, we greatly expand on previous work by characterizing population genetic structure in *B. undatum* across the North Atlantic from SNP variation obtained by RAD sequencing. There was a high degree of genetic differentiation between Canadian and European populations (Iceland, Faroe Islands, and England) consistent with the divergence of populations in allopatry (*F*_ST_ > 0.57 for all pairwise comparisons). In addition, *B. undatum* populations within Iceland, the Faroe Islands, and England are typified by weak but significant genetic structuring following an isolation‐by‐distance model. Finally, we established a significant correlation between genetic structuring in Iceland and two phenotypic traits: shell shape and color frequency. The works detailed here enhance our understanding of genetic structuring in *B. undatum* and establish the species as an intriguing model for future genome‐wide association studies.

## Graphical abstract

The current study described novel trends in population genetic structure of Common Whelk, *Buccinum undatum*, from across the North Atlantic. We described strong genetic structuring between Canadian and European populations concurrent with the divergence in allopatry, as well as associations between genotypic and phenotypic structuring in Iceland. The works detailed here enhance our understanding of genetic structuring in *B. undatum* across multiple geographical scales and establish the species as an intriguing model for future genotype–phenotype associative studies.

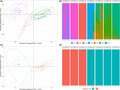

## 1 INTRODUCTION

The reconstruction of spatial genetic structure can provide valuable insights into the evolutionary processes affecting species—such as genetic drift, adaptive selection, and gene flow between populations (Crawford & Oleksiak, 2016; Funk et al., 2012; Kohn et al., 2006)—while facilitating the characterization of historical demographic events and ongoing evolutionary trajectories (Emerson et al., 2010). Marine invertebrate phylogeography in the north Atlantic Ocean has been shaped by climate variation during the Pleistocene Epoch; glacial cycles have repeatedly altered species' distributions over the last two million years. Several marine invertebrate species have gone extinct in North America and have subsequently been recolonized from Europe (Ingolfsson, 1992; Vermeij, 1991; Wares & Cunningham, 2001). Expansion from southern refugia since the last glacial maximum 18–25 k years ago is generally believed to account for present‐day distributions. However, some species survived in glacial refugia and have recolonized from more northerly latitudes (Coyer et al., 2011; Maggs et al., 2008; Panova et al., 2011), undergoing genetic bottlenecks that resulted in signatures of low genetic diversity as found in recently expanded populations.

The common whelk, *Buccinum undatum* (Linnaeus, 1758), is a commercially valuable neogastropod species characterized by slow maturation rates, a mostly sedentary adult lifestyle, internal fertilization, and direct development of larvae (Himmelman, 1988; Himmelman & Hamel, 1993; Jalbert et al., 1989; Martel et al., 1986). Common whelk is broadly distributed at high latitudes across the North Atlantic Ocean, from eastern North America to western Europe, as well as Greenland, Iceland, and the Norwegian Sea (Gendron, 1992; Golikov, 1968; Taylor & Taylor, 1977). Phylogenetic reconstructions utilizing mitochondrial (mt)DNA cytochrome oxidase subunit I (COI) markers identified two monophyletic lineages (eastern and western North Atlantic: ENA and WNA, respectively), hypothesized to have diverged early during the Pleistocene approximately 2.1 mya (Magnúsdóttir, Pálsson, Westfall, Jónsson, Goodall, et al., 2019; Pálsson et al., 2014). Molecular indices, based on COI, indicate that the WNA and ENA lineages should be considered cryptic species that have diverged in allopatry (Magnúsdóttir et al., 2019). Canadian and ENA whelk also display variation in shell morphology (Magnúsdóttir, Pálsson, Westfall, Jónsson, & Örnólfsdóttir, 2019), as well as temporal spawning differences (Canadian whelk breed in May–July while ENA whelk in September–February) (Kideys et al., 1993; Magnúsdóttir et al., 2010; Martel et al., 1986), further supporting the diversification of the Canadian and ENA lineages. Within the ENA lineage, multiple studies of *B. undatum* have reported patterns of local population structure following an isolation‐by‐distance model (Magnúsdóttir, Pálsson, Westfall, Jónsson, Goodall, et al., 2019; Mariani et al., 2012; Pálsson et al., 2014; Weetman et al., 2006) at distances as short as 30 km in Icelandic waters (Magnúsdóttir, Pálsson, Westfall, Jónsson, Goodall, et al., 2019; Pálsson et al., 2014). However, despite repeated observations of fine‐scale population structure, genetic divergence (as measured by *F*_ST_) across the entire ENA lineage is uncharacteristically low (Weetman et al., 2006). Large effective population size and recent divergence may explain the limited genetic differentiation. Semi‐continuity has also been proposed as a potential mechanism maintaining gene flow among the European populations (Mariani et al., 2012; Weetman et al., 2006).

Comparing intraspecific patterns of genotypic and phenotypic variation may also elucidate concordant or distinctive responses to environmental landscapes (Zamudio et al., 2016). Common whelk displays discordant relationships between genotypic and phenotypic variation across small geographic scales in Iceland (Magnúsdóttir et al., 2010) and Ireland (Mariani et al., 2012). In Breiðafjörður (western Iceland), shell traits such as shape and color are highly differentiated among geographically proximate populations (20–30 km), exhibiting small but significant amounts of neutral genetic variation (Magnúsdóttir, Pálsson, Westfall, Jónsson, Goodall, et al., 2019; Pálsson et al., 2014). Fine‐scale phenotypic patterns have been documented, with shell shape and color exhibiting gradients from the inner to the outer bay that correlated with environmental variables (Magnúsdóttir et al., 2018). Limited demographic connectivity was apparent from the patterns of phenotypic variation; still, with only low levels of neutral genetic differentiation among the same populations, it was hypothesized that plastic responses to environmental variation are driving the observed phenotypic divergence (Mariani et al., 2012).

Although phenotypic variation may reflect population differentiation at the molecular level, a certain level of discordance can be expected due to sampling of markers, selection on functional traits, or plasticity. Previous studies on common whelk were based on a handful of loci (microsatellite and mtDNA COI) (Magnúsdóttir, Pálsson, Westfall, Jónsson, Goodall, et al., 2019; Mariani et al., 2012; Pálsson et al., 2014; Weetman et al., 2006). Studies utilizing genome‐wide genetic datasets such as RAD sequencing (Davey & Blaxter, 2010) may be required to delineate concordant responses between the genotypic and phenotypic variations observed in common whelk. RAD sequencing has been used to resolve fine‐scale population structure in several marine invertebrates species, including the American and European lobster (Benestan et al., 2015; Jenkins et al., 2019), great and Mediterranean scallop (Vendrami et al., 2017, 2019), sea scallop (Van Wyngaarden et al., 2016), and staghorn corals (Drury et al., 2016). Additionally, RAD sequencing has the potential to detect uncharacterized cryptic species complexes within known species distributions (e.g., in the River Limpet (Weiss et al., 2017)). As such, utilization of large‐scale RAD sequencing datasets may help clarify the low overall genetic differentiation across the ENA lineage and provide further evidence of the cryptic species found within common whelk.

The current study utilizes new and extensive geographic sampling of common whelk across the North Atlantic combined with RAD sequencing technologies to assess the genetic structure within the species at varying geographic scales, ranging from a broad‐scale analysis of genetic divergence across the North Atlantic to a more targeted fine‐scale analysis of divergence within Iceland. In addition, broad‐scale analyses (herein referred to as North Atlantic‐specific analyses) aimed to evaluate the existence of cryptic species in Canada and Europe and the low genetic divergence within the ENA lineage (Magnúsdóttir, Pálsson, Westfall, Jónsson, Goodall, et al., 2019; Pálsson et al., 2014; Weetman et al., 2006). Within Iceland, individuals from Breiðafjörður Bay (western Iceland) display exceptionally high levels of shell color variation (Magnúsdóttir et al., 2018). Detailed analyses of geographical patterns of phenotypic shell traits in Breiðafjörður have been described by Magnúsdóttir et al. (2018). Therefore, in addition to describing fine‐scale population trends in Breiðafjörður using a RAD sequencing approach, we aim to examine further the relationship between population genetic differentiation and existing phenotypic profiling data (shell color and shape) within the bay.

## 2 MATERIALS AND METHODS

### 2.1 Sample collection

**Figure 1:**
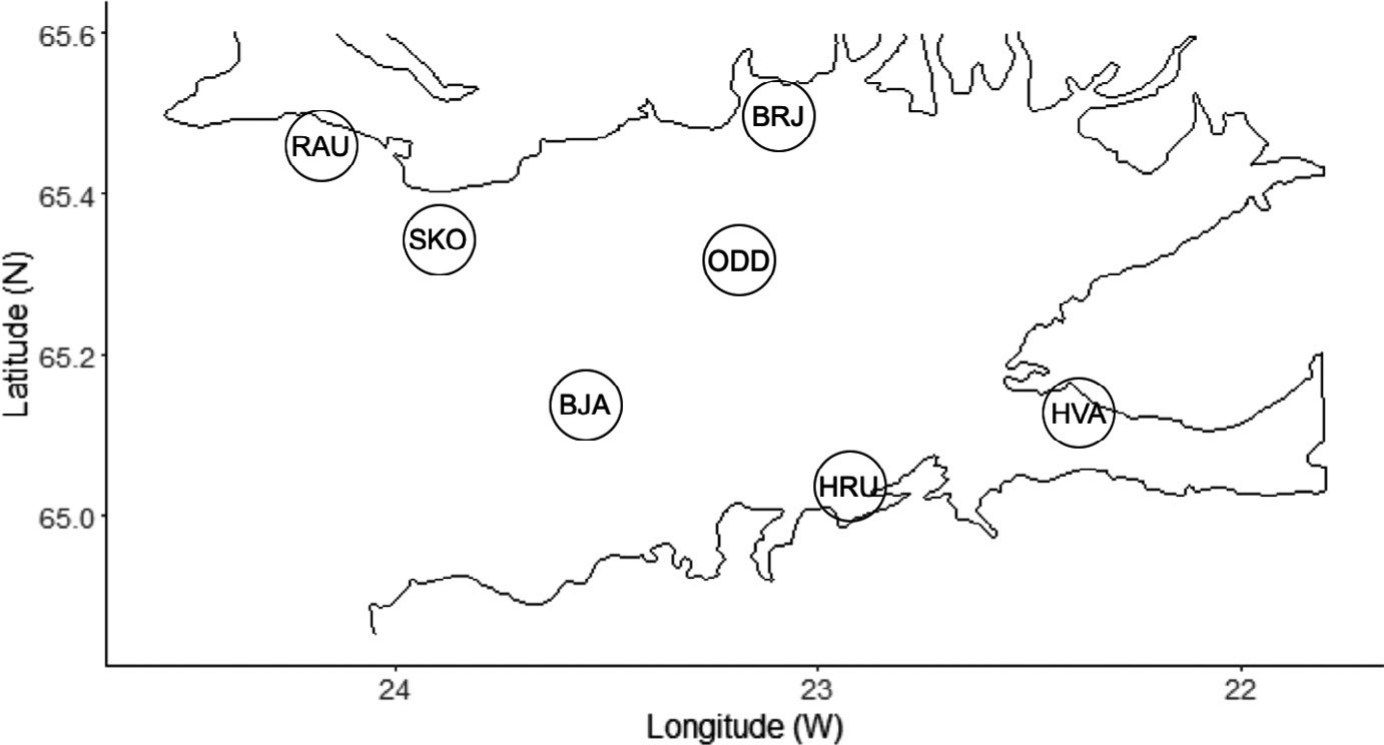
Sampling sites across the Bay of Breiðafjörður, west Iceland. *Buccinum undatum* were collected from the following sites: Rauðasandur (RAU), Skor (SKO), Bjarneyjaráll (BJA), Oddbjarnarsker (ODD), Brjánslækur (BRJ), Hrútey (HRU), and Hvammsfjörður (HVA). GPS coordinates for each sample site can be found in Table 1

**Figure 2:**
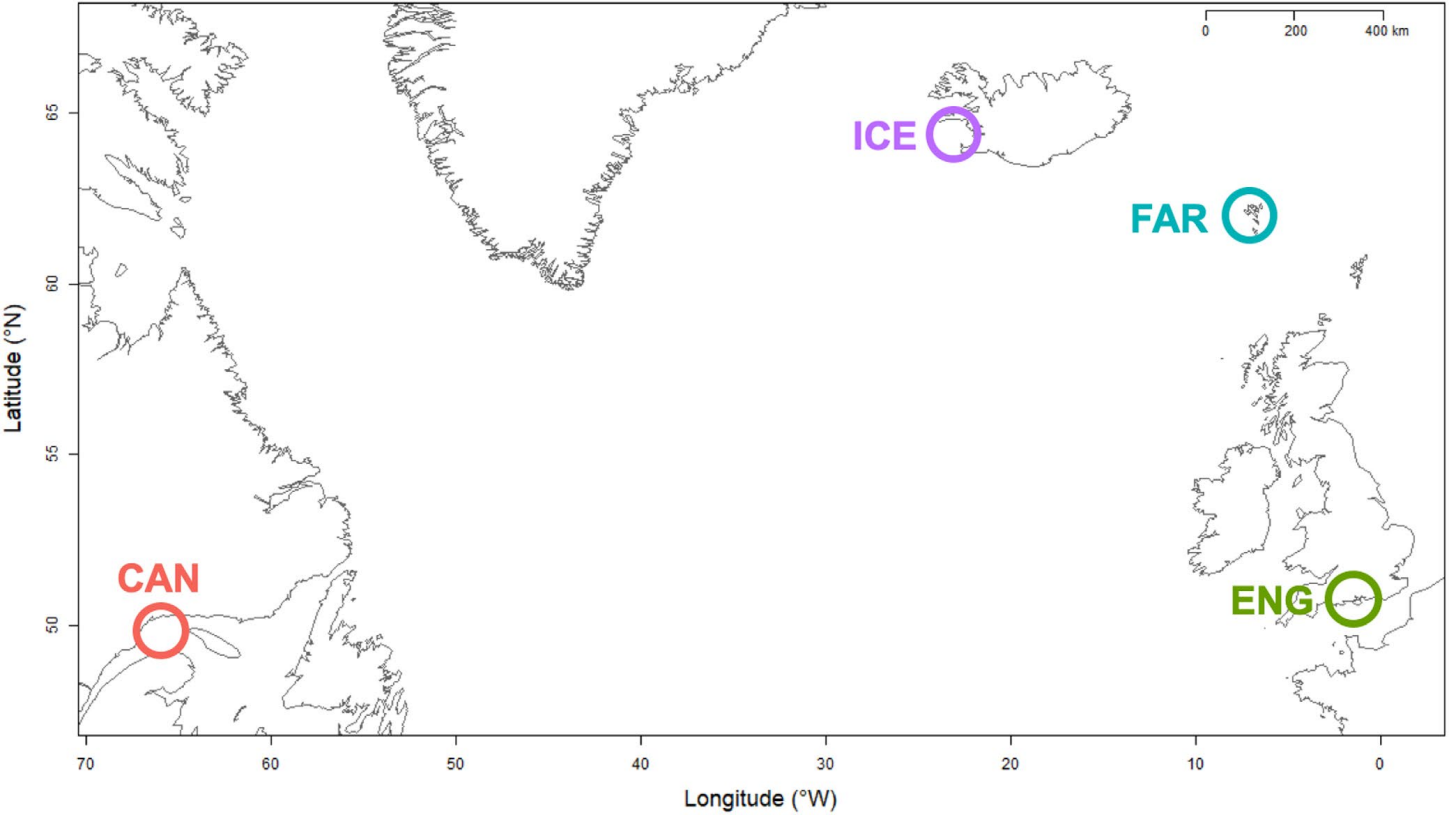
Distribution of *Buccinum undatum* sample sites across the North Atlantic. Individuals sampled are representative of populations from Canada, the Gulf of Saint Lawrence (CAN), Faroe Islands (FAR), England (ENG), and Breiðafjörður in west Iceland. GPS coordinates for each sample site can be found in Table 1

Live *B. undatum* were collected at seven sites in Breiðafjörður, Iceland (IS, *n* = 295, Figure 1): Bjarneyjaráll (BJA, *n* = 31), Brjánslækur (BRJ, *n* = 60), Hvammsfjörður (HVA, *n* = 58), Hrútey (HRU, *n* = 66), Oddbjarnarsker (ODD, *n* = 40), Skor (SKO, *n* = 32), and Rauðasandur (RAU, *n* = 8). Across the North Atlantic (Figure 2), whelk were sampled from Canada (CAN, Quebec, Gulf of St. Lawrence, *n* = 29)*,* England (ENG, English Channel, *n* = 30), and Faroe Islands (FAR, *n* = 29). Geographical coordinates for all sample sites are listed in Table 1. For all individuals, foot tissue was sampled via dissection and fixed immediately in 96% ethanol. Samples were stored at 4°C for 2 days before being exchanged with fresh ethanol and stored at –30°C.

**Table 1:** Sample locations, depths, GPS coordinates, and sample numbers (pre‐ and post‐data filtering) for *Buccinum undatum* sampled within Breiðafjörður (Iceland) and across the North Atlantic (Canada, England, Faroe Islands)

Location	Abbreviation	Depth (m)	Latitude	Longitude	Year Sampled	Captured (*n*)	Analyzed (*n*)
Iceland	IS	–	–	–	2014	295	104
Bjarneyjaráll	BJA	125	65° 08′27″ N	23° 35′04″ W	2014	31	9
Brjánslækur	BRJ	37	65° 29′52″ N	23° 08′24″ W	2014	60	9
Hvammsfjörður	HVA	15	65° 07′36″ N	22° 22′37″ W	2014	58	34
Hrútey	HRU	36	65° 01′34″ N	22° 56′20″ W	2014	66	18
Oddbjarnarsker	ODD	43	65° 18′50″ N	23° 14′01″ W	2014	40	4
Skor	SKO	53	65° 20′26″ N	23° 55′28″ W	2014	32	24
Rauðasandur	RAU	31	65° 27′59″ N	24° 09′05″ W	2014	8	6
Canada	CAN	18	50° 02′24″ N	66° 25′48″ W	2015	29	28
England	ENG	10	50° 23′24″ N	1° 22′12″ W	2007	30	14
Faroe Islands	FAR	40	61° 46′48″ N	7° 36′00″ W	2008	29	18

### 2.2 DNA extraction, RAD library preparation, and sequencing

Fixed foot tissue was dried briefly (to evaporate residual ethanol) and cut into small pieces that underwent DNA isolation using an Omega Bio‐Tek E.Z.N.A. Mollusc DNA Kit (as per manufacturer's instructions, https://www.omegabiotek.com/product/e‐z‐n‐a‐mollusc‐dna‐kit/). Double digest RAD sequencing libraries were constructed by digesting whole genomic DNA with *Sau*3AI and *Ape*KI and selecting fragments 390 bp to 430 bp using a Pippin Prep (Sage Science). Individuals were assigned a molecular identification tag using combinatorial barcoding of forward (5bp) and reverse (6bp) reads, respectively, then pooled into four separate libraries before size selection and amplification using 10 PCR cycles. Resulting library concentrations were quantified using SYBR Gold double‐stranded DNA assay measured on a TECAN GENios plate reader (TECAN^™^, www.tecan.com), and the sizes estimated by 2% agarose gel. Library quality was assessed using two pilot sequencing runs on Illumina MiSeq (paired‐end 2 × 150 bp). Libraries were sequenced using Illumina HiSeq 2500 (paired‐end 2 × 125 bp) across four lanes, generating an average of 4 ± 3.5 *SD* million paired raw reads per individual.

### 2.3 Data assessment, quality filtering, and stacks assembly

Raw reads were demultiplexed in paired mode (*‐‐paired*) using the *process_radtags* program in *Stacks* v.2.0b (Catchen et al., 2011, 2013), with restriction enzymes specified (*‐‐renz_1 apeKI*; *renz_2 sau3AI*) and the *‐‐inline_inline*, *–r*, *–c*, *–q*, *–t* *=* *119* options enabled (demultiplexed reads available from Goodall, et al., 2020). Note that reads were truncated to 119 bp to ensure equal length following the removal of molecular identification tags. Demultiplexed reads (see Table S1), which included both Illumina MiSeq and HiSeq data (average of 3,499,689 (±3,131,707 standard deviation) reads per sample), were processed using the de novo *stacks* pipeline (i.e., *ustacks*, *cstacks*, *sstacks*, *tsv2bam*, *gstacks*, and *populations*).

The initial pass through the de novo *stacks* pipeline was designed to optimize the sample selection of downstream primary analyses by identifying and removing low‐quality individuals within the dataset. Overall, *Stacks* parameters were selected with the aim of retaining a high number of SNPs, with more rigorous filtering of SNPs and individuals occurring downstream. Initially, trimmed reads were processed in *ustacks* with the *–M 4*, *–m 3* options enabled. To generate *cstacks* catalogs, sample inputs were limited to *n* = 200 individuals, following the recommendations of Rochette and Catchen (2017). To avoid the inclusion of over‐ or under‐sequenced individuals in *cstacks* assemblies, the median total read count (as derived from FastQC (Andrews, 2010)) was calculated from all *n* = 383 individuals. The *n* = 200 individuals used constituted those with a total read count closest to this median value, which included *n* individuals from each of the four North Atlantic sample locations: Canada(n) = 19; England(n) = 14; Faroe Island(n) = 17; Iceland(n) = 150. Following *cstacks* catalog construction (*–n 6* option enabled), the remaining de novo *stacks* pipeline proceeded using the original *n* = 383 samples. *Sstacks* and *tsv2bam* steps proceeded using default settings, while *gstacks* was run in de novo mode (*‐P* option enabled). Finally, *populations* analyses were run with *–p* 3 and *–r* 0.5 options enabled, where the *‐‐popmap* designated individuals based on their country of origin (i.e., Canada, England, Faroe Islands, and Iceland).

The resulting *populations* .vcf file was imported into *Radiator* (Gosselin, 2017) for quality analysis. Radiator's *filter_rad* pipeline was run in interactive mode, with the aim of blacklisting poor‐quality markers and individuals. Filtering parameters and outputs are listed in Table S2. After *Radiator* filtering, the following samples were considered as whitelisted and used for all primary analyses downstream: Canada(n) = 28; England(n) = 14; Faroe Island(n) = 18; and Iceland(n) = 117.

Primary analyses were initiated by undertaking a second pass through the de novo *stacks* pipeline using only the optimized *n* = 177 whitelisted sample set. Again, *Stacks* parameters were selected with the aim of retaining high numbers of SNPs, with more rigorous filtering occurring downstream in *Radiator*. Initially, the *cstacks* catalog was reassembled (*–n 6* option enabled) using all *n* = 177 individuals. *Sstacks* and *tsv2bam* steps were run using default settings, and *gstacks* run in de novo mode (*–P* option enabled), yielding 900,825 loci, with an effective per‐sample coverage of 17.7 (± 6.8 standard deviation). *Populations* analyses were again run with *–p* 3 and *–r* 0.5 options enabled, and *‐‐popmap* designating individuals based on their country of origin. Low‐quality individuals and markers were again filtered using *Radiator* (parameters and outputs listed in Table S3). The number of individuals retained, postfiltering, across all geographic scales of analysis is listed in Table 1. The average coverage per marker following *Radiator* was 18.6 (±6.3 standard deviation).

Departure from Hardy–Weinberg equilibrium (HWE) was assessed using a chi‐squared test (*χ^2^) of expected genotype frequencies in the R‐package Pegas* (Paradis, 2010), where the false discovery rate of *p* was controlled for using Benjamini and Yekutieli (2001) procedure for multiple testing. HWE tests were undertaking in a population‐wise manner, where population status was assigned based on country of origin, except for Icelandic individuals who were assigned population status relative to their sample site of origin in Breiðafjörður. In addition to population‐wise chi‐squared values, combined probabilities across all populations were calculated using Fisher's combined probability (Sokal & Rohlf, 1969) and adjusted using Benjamini‐Yekutieli's procedure. Loci were removed from the dataset when either two or more populations significantly deviated from HWE, or significant Fisher's combined probability values (*p* < .05) were observed (these parameters are not mutually exclusive). Notably, departure from HWE was assessed on a scale‐specific manner; firstly, at the North Atlantic scale where all individuals from all populations (with all sites from Breiðafjörður representing Iceland) were included and, secondly, at the Icelandic scale where only individuals from Iceland were assessed (again with population designated relative to sample site in Breiðafjörður). Adaptive SNPs (putative loci under selection) were identified either at the North Atlantic or Icelandic scale using *BayeScan* (Foll & Gaggiotti, 2008), with parameters set for 50,000 iterations with prior odds of 100, following 20 pilot runs of 5,000 interactions each, a thinning interval of 10, and a burn‐in length of 50,000. Confirmation of Markov chain convergence was conducted using Gelman and Rubin multiple sequence diagnostic and Heidelberger and Welch's Convergence diagnostic tests within the *R*‐package *CODA* (Plummer et al., 2006). Putative adaptive SNPs were removed from the North Atlantic or Icelandic datasets using *Adegenet* (Jombart & Ahmed, 2011) (loci were considered outliers under a false discovery rate (FDR) of 0.05). All downstream analysis of the quality filtered North Atlantic‐ and Iceland‐specific datasets were conducted independently.

### 2.4 Analysis of genetic variation and population divergence

The estimation of genetic variation and divergence was undertaken in both North Atlantic and Icelandic datasets using the neutral SNP dataset exclusively. Mean observed heterozygosity (*H*_O_), expected heterozygosity (*H*_E_), and inbreeding coefficient (*F*_IS_) per population were calculated using *Hierfstat* (Goudet, 2005). All pairwise *F*_ST_ calculations were computed in *DartR* following 1,000 permutations (Gruber et al., 2018). The presence of putative population clusters (*K*) within the genomic data was estimated using the *find.clusters* function of *Adegenet* (Jombart, 2008; Jombart & Ahmed, 2011). The optimal number of clusters (*K*) was selected by computing the value of *K* with the lowest associated goodness of fit value estimated with BIC.

Population genetic structure was visualized by using Gower PCoA ordination (using Euclidean distance) in *DartR*. Population structure was visualized using PCoA both when considering population assignments as sample site of origin or as a function of the putative population clusters (*K*) assignments. Population admixture was estimated using the Discriminant Analysis of Principal Components (DAPC) in *Adegenet* (Jombart et al., 2010). The number of principal components retained within DAPC was optimized using the a‐score optimization test prior to analysis. Admixture was expressed both when considering population assignments as sample site of origin or as a function of the putative population clusters (*K*) assignments. Isolation‐by‐distance was assessed by analyzing the association of genetic and geographic distances for all individuals using a Mantel test in *Ade4* (Dray & Dufour, 2007; Mantel, 1967). Relative migration rates for Icelandic individuals were calculated on a per‐sample site basis using the *divMigrate* function in *diveRsity* (Keenan et al., 2013), where relative migration was derived from Nei's G_ST_ statistic.

Finally, genomic divergence across the North Atlantic was investigated by assaying haplotype coancestry. *Populations* derived haplotype.tsv files were converted to a *fineRADstructure* (Malinsky et al., 2018) compatible format using *fineRADstructure_tools* (https://github.com/edgardomortiz/fineRADstructure‐tools). Haplotype coancestry of North Atlantic populations was calculated using *fineRADstructure* and visualized in *R* using the scripts provided in the *fineRADstructure* manual (http://cichlid.gurdon.cam.ac.uk/fineRADstructure.html).

### 2.5 Comparison of genetic and phenotypic trends in Breiðafjörður, Iceland

Comparisons of genetic and phenotypic distances were performed for the Icelandic dataset only. Phenotypic data corresponding to each of the Icelandic sites was sourced from Magnúsdóttir et al. (2018). No phenotypic data could be sourced from RAU (Rauðasandur), and therefore, individuals from this site were excluded from phenotype association analyses. Phenotypic data corresponding to the following measures were sourced: (a) shell shape, based on eleven geometric morphometric landmarks on the ventral surface of the shell that combine information on shell spire/body whorl ratio, aperture shape, shell shape, and shell lip thickness; and (b) shell color frequency, based on the primary color of the shell (See Table S4).

Correlation of genetic distances (euclidean) with phenotypic distances was tested on a per‐sample site basis, using Mantel's test, with 1,000 permutations in the R‐package *vegan* (Oksanen et al., 2017). Euclidean distances were calculated for variation in shell shape between sites, while differences in shell color composition at each site were assessed using Bray–Curtis distances (Oksanen et al., 2015). To account for spatial autocorrelation due to either geographic distance or differences in depth, a partial Mantel test was implemented (Smouse et al., 1986) whereby either the effect of geographic distance or depth differences was kept constant.

The relationship between population genetic and phenotypic structuring was tested using cluster (*K* = 2) population assignments. Correlation between putative population clusters and shell shape differences were assessed using permutational multivariate analysis of variance of shape distances, implemented using the *adonis*() function in *vegan* (Oksanen et al., 2017) with the assigned putative genetic clusters as the explanatory variable. Similarly, correlation between putative population clusters and shell color were assessed by comparing differences in shell color frequencies between putative population clusters, using Fisher's exact test (Fisher, 1934).

## 3 RESULTS

### 3.1 Population genetic structure and divergence across the North Atlantic

Following genotype calling (*populations*) and *Radiator* quality filtering, North Atlantic‐specific analyses (*n* = 164 individuals) yielded a total of 35,836 common putative single locus SNP across Canada (*n* = 28), Iceland (*n* = 104), Faroe Islands (*n* = 18), and England (*n* = 14). A further *n* = 314 loci significantly deviated from HWE and were removed from the dataset, as were *n* = 2 loci flagged as putative adaptive SNP candidates (qval ≤ 0.05). All downstream analyses were performed on the remaining *n* = 35,518 putative neutral loci (total missing data 18.4%) retained postfiltering.

**Figure 3:**
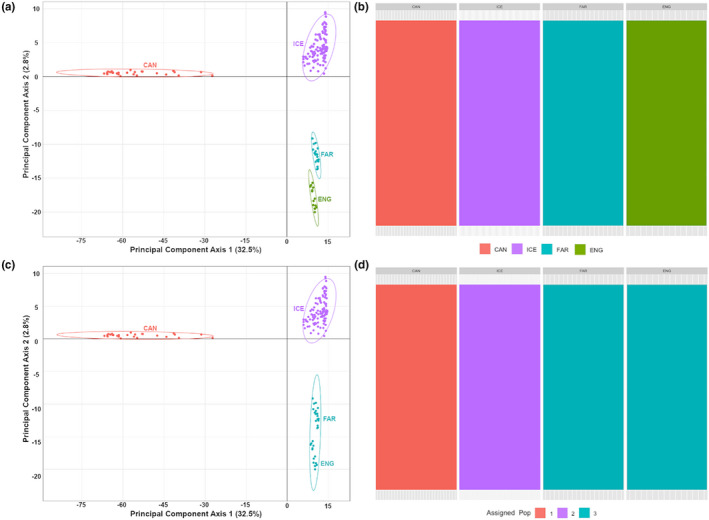
Summary of inferred population genetic structure for *Buccinum undatum* (*n* = 164) sampled from across the North Atlantic. All plots are derived from 35,518 putatively neutral SNP. Plots corresponding to the following: (a) Gower PCoA ordination (using euclidean distance) plot where population is assigned relative country of origin; (b) Admixture plot where population is assigned relative country of origin; (c) Gower PCoA ordination plot where population is assigned relative to optimal cluster (*K* = 3) assignments; (d) Admixture plot where population is assigned relative to optimal cluster (*K* = 3) assignments. Individuals are labeled relative country of origin: Canada (CAN, orange), Iceland (ICE, purple), Faroe Island (FAR, blue), and England (ENG, green)

Genetic diversity was similar in all ENA populations (Iceland, Faroe Islands, England), with observed heterozygosity ranging from 0.146 to 0.158 (Table 2), while gene diversity was lowest for Canadian individuals (*H*_O_ = 0.107). None of the North Atlantic populations displayed signals of inbreeding with *F*_IS_ ranging from 0.043 to 0.068. At the North Atlantic scale, calculations of pairwise *F*_ST_ identified significant genetic divergence for all pairwise comparisons (*p* < .001, Table 3). *F*_ST_ values for all pairwise comparisons with Canadian exceeded 0.57, while *F*_ST_ values within the ENA ranged from 0.059 to 0.098. Overall, genetic divergence followed a model of isolation‐by‐distance (*r* *=* 0.884, *p* *=* .001), with the Faroe Islands and England whelk displaying the greatest degree of genetic similarity, followed by Iceland, while Canada represented the most genetically divergent population relative to ENA populations (Figure 3a). No signals of population admixture (DAPC optimized to retain *n* = 23 principal components) were observed between Canada, Iceland, Faroe Islands, and England (Figure 3b).

**Table 2:** Summary of average observed heterozygosity (H_O_), expected heterozygosity (H_E_), and inbreeding coefficient (F_IS_) for *Buccinum undatum* populations sampled from across the North Atlantic. Populations are designated relative to the country of origin with countries denoted as CAN (Canada), ICE (Iceland), FAR (Faroe Island), and ENG (England). The standard error is denoted as SE

Population	Individuals	*H*_O_	±SE	*H*_E_	±SE	*F*_IS_	±SE
CAN	28	0.107	0.001	0.108	0.001	0.047	0.001
ICE	104	0.157	0.001	0.167	0.001	0.068	0.001
FAR	18	0.158	0.001	0.159	0.001	0.043	0.001
ENG	14	0.146	0.001	0.148	0.001	0.050	0.001
Overall	164	0.147	0.001	0.214	0.001	0.053	0.001

**Table 3:** Pairwise *F*_ST_ values for *Buccinum undatum* sampled across the North Atlantic. Values of *F*_ST_ are listed above the diagonal, while values below represent significance values (*p*) of pairwise comparisons. Significance values (*p*) of pairwise comparisons were obtained following 1,000 permutations. Populations are listed based on country of origin, with abbreviations corresponding to the following: CAN (Canada); ICE (Iceland); ENG (England); and FAR (Faroe Islands)

	CAN	ICE	FAR	ENG
CAN	–	0.572	0.603	0.619
ICE	<0.001	–	0.061	0.098
FAR	<0.001	<0.001	–	0.059
ENG	<0.001	<0.001	<0.001	–

The Bayesian information criterion estimated optimal goodness of fit value at *K* = 3 population clusters. Canadian and Icelandic populations formed distinct putative populations relative to the Faroe Islands and England whelk (Figure 3c). No signals of population admixture were detected between putative population assignments at the North Atlantic (DAPC optimized to retain *n* = 23 principal components).

**Figure 4:**
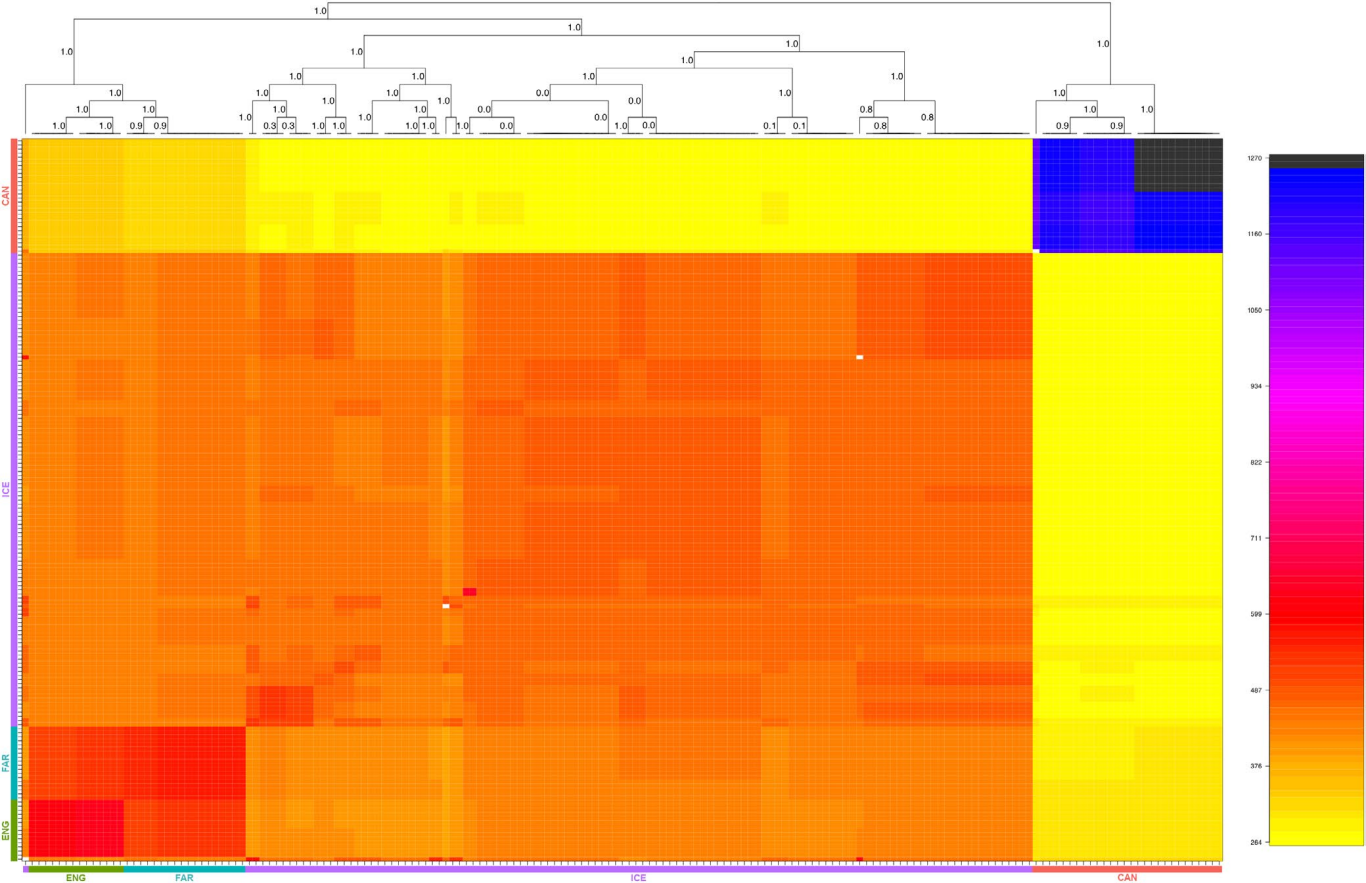
Haplotype coancestry matrix inferred for North Atlantic *Buccinum undatum* populations. Each pixel within the heatmap represents the inferred coancestry coefficient for a given individual based on haplotype loci. Colored bars adjacent to the heatmap represent individuals' country of origin, including England (green), the Faroe Islands (blue), Iceland (purple), and Canada (red). Within the heatmap, individuals with the highest shared coancestry are depicted in darker colors, while lesser values of coancestry are depicted in brighter shades. Bootstrap support is listed for each branch divergence for the inferred phylogeny

Haplotype coancestry analyses indicated a lack of shared genetic ancestry between CAN and all ENA individuals, with the split of CAN and the ENA lineages occurring before any subsequent ENA divergence (Figure 4). Regarding the ENA clade, Icelandic individuals formed an outgroup distinct from the Faroe Islands and English haplotypes. Within the Iceland‐specific branch, haplotypes were primarily structured into two further subgroups. For the Faroe Island‐ and England‐specific clade, individual haplotypes were structured by country of origin, with Faroe Islands and English individuals forming two distinct sister groups.

### 3.2 Population genetic structure and divergence in Iceland

Quality filtering reduced sample representation across all sample sites (particularly for ODD), with Iceland‐specific analyses being conducted across BJA (*n* = 9), BRJ (*n* = 9), HVA (*n* = 34), HRU (*n* = 18), ODD (*n* = 4), SKO (*n* = 24), and RAU (*n* = 6). Following the preparation of the Icelandic‐specific dataset (35,836 common putative single locus SNP across all individuals), quality filtering removed a further *n* = 268 loci, which significantly deviated from HWE, as well as a further *n* = 175 putatively adaptive SNP candidates (qval ≤ 0.05). All downstream Iceland‐specific analyses proceeded with the remaining *n* = 35,393 putative neutral loci (total percentage of missing data = 19.4%).

**Figure 5:**
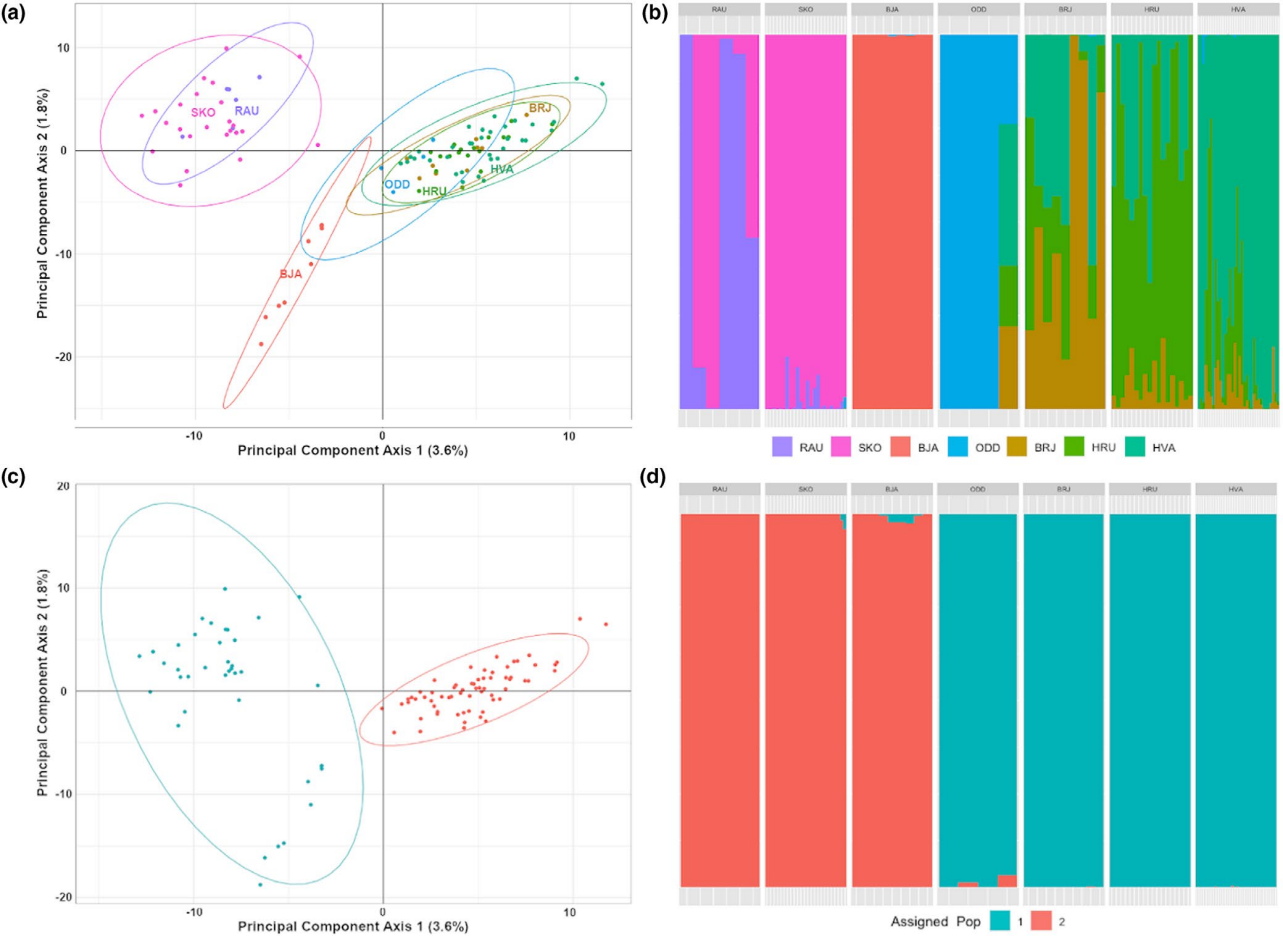
Summary of inferred population genetic structure for *Buccinum undatum* (*n* = 104) sampled from Breiðafjörður, Iceland. All plots are derived from 35,836 putatively neutral SNP. Plots corresponding to the following: (a) Gower PCoA ordination (using euclidean distance) plot where individual population is assigned relative to capture site in Breiðafjördur; (b) Admixture plot where individual population is assigned relative to capture site in Breiðafjördur; (c) Gower PCoA ordination plot where individual population is assigned relative to optimal cluster (*K* = 2) assignments; (d) Admixture plot where individual population is assigned relative to optimal cluster (*K* = 2) assignments. Individuals are labeled relative to capture site in Breiðafjördur: Rauðasandur (RAU, purple), Skor (SKO, pink), Bjarneyjaráll (BJA, red), Oddbjarnarsker (ODD, blue), Brjánslækur (BRJ, brown), Hrútey (HRU, green), and Hvammsfjörður (HVA, blue‐green)

**Figure 6:**
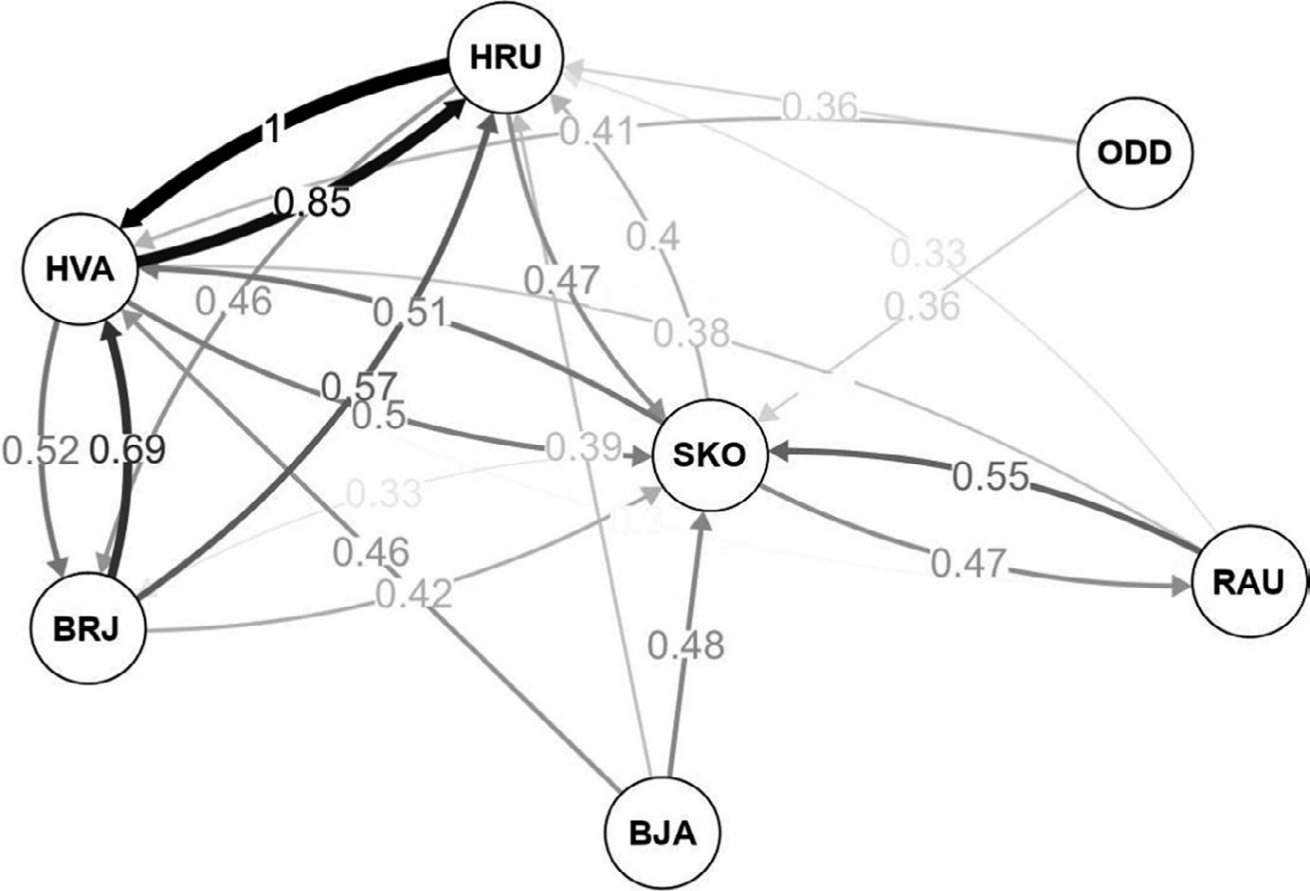
Relative migration network of sample sites within Breiðafjörður, Iceland. Migration rates are calculated from Nei's GST. Filter thresholds were set to exclude weak migration patterns at values < 0.3. Sample sites within Breiðafjörður are abbreviated as follows: Rauðasandur (RAU), Skor (SKO), Bjarneyjaráll (BJA), Oddbjarnarsker (ODD), Brjánslækur (BRJ), Hrútey (HRU), and Hvammsfjörður (HVA)

Genetic diversity was similar across all Icelandic populations, with observed heterozygosity ranging from 0.149 to 0.165 and inbreeding coefficient (*F*_IS_) ranging from −0.019 to 0.040 (Table 4). Calculations of pairwise *F*_ST_ identified significant genetic divergence between all pairwise comparisons (*p* *<* .001, Table 5). Genetic divergence followed a model of isolation‐by‐distance (*r* = 0.203, *p* = .001), with individuals within the outermost (SKO, RAU, BJA) and innermost (BRJ, HRU, HVA, ODD) regions of Breiðafjörður, generally, displaying the greatest degree of genetic similarity (Figure 5a) and admixture (Figure 5b), respectively. Estimations of clusters (K) in *adegenet* indicated further regional segregation of population genetic variation, predicting an optimal BIC goodness of fit value at *K* = 2, with clusters segregating outer‐ (SKO, RAU, BJA) and innermost (BRJ, HRU, HVA, ODD) bay sites (Figure 5c). When considering the optimal cluster (*K*) population assignments, admixture plots indicated minimal gene flow between the putative outer‐ and innermost bay populations (see Figure 5d). Migration analyses indicated that coastal sites generally share the greatest degree of migration (Figure 6), particularly for the inner bay sites BRJ, HRU, and HVA. For both mid‐bay sites (BJA and ODD), which occur at the greatest depth, unidirectional migration patterns were observed, with migration occurring exclusively from deep site to proximal coastal sites.

**Table 4:** Summary of average observed heterozygosity (*H*_O_), expected heterozygosity (*H*_E_), and inbreeding coefficient (*F*_IS_) for *Buccinum undatum* populations sampled from Breiðafjörður Bay, Iceland. Populations are designated relative to their sample site of origin with sites denoted as BJA (Bjarneyjaráll), BRJ (Brjánslækur), HVA (Hvammsfjörður), HRU (Hrútey), ODD (Oddbjarnarsker), SKO (Skor), and RAU (Rauðasandur). The standard error is denoted as SE

Population	Individuals	*H*_O_	±SE	*H*_E_	±SE	*F*_IS_	±SE
BJA	9	0.149	0.001	0.140	0.001	0.026	0.001
BRJ	9	0.157	0.001	0.148	0.001	0.016	0.001
HRU	18	0.157	0.001	0.157	0.001	0.040	0.001
HVA	34	0.160	0.001	0.162	0.001	0.034	0.001
ODD	4	0.165	0.001	0.143	0.001	−0.019	0.002
RAU	6	0.161	0.001	0.150	0.001	0.017	0.002
SKO	24	0.159	0.001	0.161	0.001	0.036	0.001
Overall	104	0.158	0.001	0.167	0.001	0.024	0.001

**Table 5:** Pairwise *F*_ST_ values for Buccinum undatum sampled within Breiðafjörður Bay, Iceland. Values of *F*_ST_ are listed above the diagonal, while values below represent significance values (*p*) of pairwise comparisons. Significance values (*p*) of pairwise comparisons were obtained following 1,000 permutations. Populations are listed relative to individuals sample site of origin, with abbreviations corresponding to the following: BJA (Bjarneyjaráll), BRJ (Brjánslækur), HVA (Hvammsfjörður), HRU (Hrútey), ODD (Oddbjarnarsker), SKO (Skor), and RAU (Rauðasandur)

	BJA	BRJ	HRU	HVA	ODD	RAU	SKO
BJA	–	0.068	0.067	0.068	0.051	0.057	0.053
BRJ	<0.001	–	0.009	0.007	0.015	0.035	0.038
HRU	<0.001	<0.001	–	0.007	0.012	0.036	0.040
HVA	<0.001	<0.001	<0.001	–	0.012	0.035	0.038
ODD	<0.001	<0.001	<0.001	<0.001	–	0.018	0.026
RAU	<0.001	<0.001	<0.001	<0.001	<0.001	–	0.003
SKO	<0.001	<0.001	<0.001	<0.001	<0.001	<0.001	–

### 3.3 Comparison of genetic and phenotypic variation in Iceland

No significant correlation between shell shape distances or color dissimilarity were observed (*p* > .05) when considering individuals on a site‐specific basis, regardless of correction for spatial autocorrelation (geographic distance or depth). Sites were grouped into putative population clusters based on K‐means clustering (*K* = 2) and analyzed for correlations between genetic distance and phenotypic traits, shell shape distances, and color frequency (Magnúsdóttir et al., 2018). Significant differences in both color frequency (Fisher's test: *p* = .0005) and shape distances (*F*′ = 15.95, *p* = .001) were observed between the two putative genetic clusters. The outer bay cluster consisted primarily of lighter shell colors, with orange and white being the most common (Table S4), while the inner bay cluster consisted predominantly of green and brown shells. With regard to shell shape, the outer bay cluster was correlated with rounder shells, a proportionally shorter spire (compared with the body whorl), and a more elongate aperture (Magnúsdóttir et al., 2018), whereas the inner bay cluster was correlated with more elongate shells, with a comparatively taller spire, and smaller rounder apertures.

## 4 DISCUSSION

The current study undertook a comprehensive analysis of North Atlantic population genetic structure in the common whelk utilizing a RAD sequencing approach. WNA (Canada) and ENA (England, Faroe Islands, and Iceland) populations were highly divergent, with an *F*_ST_ ≥ 0.57 relative to within‐ENA divergence (*F*_ST_ ≤ 0.1), bolstering previous claims that the two lineages constitute cryptic species that diverged under allopatric speciation (Magnúsdóttir, Pálsson, Westfall, Jónsson, Goodall, et al., 2019; Pálsson et al., 2014). Smaller but significant differentiation was also observed between all ENA sites (Iceland, Faroe Islands, England), with putative cluster assignments suggesting genetic substructuring within the ENA likely exists, particularly regarding Icelandic populations being differentiated from both Faroe Islands and English populations. Haplotype coancestry analyses further support the characterization of ENA populations as a distinct lineage, typified by moderate to low intra‐lineage population divergence. Limited but significant population structure following an isolation‐by‐distance model was also detected in Icelandic populations, suggesting that limited dispersal in *B. undatum* constrains gene flow at localized and broad geographic scales across the ENA. Analyses of genetic variation in the bay of Breiðafjörður (Iceland) identified two putative population clusters composed exclusively of individuals sampled from the bay's outer‐ and innermost regions. Significant correlation between shell shape variation and shell color frequency were observed in Iceland, with phenotypic traits showing concordance with the denotation of Breiðafjörður whelk as distinct outer‐ and innermost bay population clusters.

Direct‐developing, benthic marine species are hypothesized to exhibit greater population genetic structure, relative to broadcast spawners, due to their limited dispersal capabilities. While rafting of egg masses may confer some means of dispersal for direct‐developing gastropods (see Johannesson, 1988; Kyle & Boulding, 2000; Marko, 2004), the frequency of such events is unlikely to maintain gene flow to an extent which counteracts the effects of high self‐recruitment. Existing population genetic studies of *B. undatum* described genetic structuring following an isolation‐by‐distance model across the North Atlantic (Magnúsdóttir, Pálsson, Westfall, Jónsson, Goodall, et al., 2019; Mariani et al., 2012; Pálsson et al., 2014; Weetman et al., 2006); however, it remained unclear whether the variation observed from mitochondrial/microsatellite markers was representative of broader genomic trends. In the present study, trends in population structure both within Iceland and across the North Atlantic (as scored from a set of RAD sequencing SNPs) were commensurate with existing mitochondrial/microsatellite works.

Our results reaffirm mtDNA data at the North Atlantic scale, indicating the Canadian and ENA populations constitute two distinct genetic lineages, which diverged some 2.1 million years ago (Magnúsdóttir, Pálsson, Westfall, Jónsson, & Örnólfsdóttir, 2019). Haplotype coancestry estimates indicated Canadian and ENA whelk represent two distinct genetic lineages, with subsequent divergence within the ENA lineage occurring more recently. For all pairwise comparisons, *F*_ST_ and DAPC analyses indicated significant genetic divergence with restricted gene flow between Canadian and ENA populations. Recent work focused on the *B. undatum* mtDNA gene, *COI*, indicated the presence of cryptic species or clear allopatric divergence between Canadian and ENA whelk based on multiple species screening indices (Magnúsdóttir, Pálsson, Westfall, Jónsson, & Örnólfsdóttir, 2019). Regarding SNP‐based population genetic comparisons, Hey and Pinho (2012) proposed an *F*_ST_ = 0.35 as the upper threshold for intra‐species population comparisons, whereby comparisons exceeding this value likely constitute interspecies comparisons. Under this scheme, Canadian and the ENA whelk exceed Hey and Pinho's (2012) *F*_ST_ threshold at ≥0.57 and would thus be considered separate species. Nevertheless, while our data further consolidate evidence of substantial genetic divergence between Canadian and ENA lineages, breeding studies are ultimately required to determine whether the two lineages represent reproductively isolated species conforming to the biological species concept. However, due to asynchronous breeding times, these populations are unlikely to interbreed in nature.

Our results support claims of low, but significant, genetic divergence as a persistent feature within the ENA lineage. Further research is required to derive the mechanisms driving low divergence within the ENA; however, our data suggest that maintenance of gene flow under a stepping stone model is unlikely given little to no admixture is observed between Icelandic, Faroe Islands, and English whelk. At the Icelandic scale, our estimates of *F*_ST_ (ranging from 0.003 to 0.068) fall within the ranges previously estimated for Breiðafjörður using mtDNA (ranging from 0.0003 to 0.145) (Pálsson et al., 2014). On a regional basis, global *F*_ST_ for Iceland (global *F*_ST_ = 0.029) is similar, albeit higher on average, to those derived for British (global *F*_ST_ = 0.014) and Irish (global *F*_ST_ = 0.019) *B. undatum* populations (Mariani et al., 2012; Weetman et al., 2006).

While previous studies have speculated on the occurrence of multiple spatially differentiated *B. undatum* populations within Breiðafjörður (Magnúsdóttir et al., 2010, 2018; Woods & Jonasson, 2017), our study is the first to characterize fine‐scale genetic structure in the region. Interestingly, profiles of heterozygosity and genetic divergence in Iceland are dissimilar to those described for Britain. Weetman et al. (2006) characterized reduced genetic diversity and greater genetic differentiation in inshore, relative to offshore populations, resulting from unidirectional migration by British whelk. Breiðafjörður's populations exhibit similar observed heterozygosity across all site‐specific populations, with populations from the outermost regions of Breiðafjörður (BJA, RAU, and SKO) displaying the greatest degree of genetic differentiation. To summarize, trends in genetic diversity and divergence in Iceland do not indicate unidirectional offshore migration, as seen in Britain. Instead, modeling of migration patterns indicated that some degree of migration likely occurs between all sites; however, said migration is primarily restricted to shallower more coastal sites and, even then, occurs mostly within the putatively assigned outermost and innermost bay population clusters (a trend also reflected by estimations of admixture).

Interestingly, both BJA and ODD, which represent the deepest sites for each putative population cluster, respectively, displayed unidirectional migration patterns toward shallower more coastal sites. Signatures of restricted gene flow in both BJA and ODD were evident in site‐specific admixture plots, even within their putatively assigned population clusters. Shallower, more coastal sites may be more susceptible to disturbance (i.e., in the case of storms), increasing the frequency of rafting events, and thus, admixture between coastal sites. Surface waters in Breiðafjörður are considered to be well mixed, with nearshore currents flowing in a continuous clockwise fashion (Logemann et al., 2013) across Breiðafjörður. Taken together, deeper sites such as BJA and ODD are likely more stable (with respect to storm events) and less influenced by coastal currents, resulting in reduced gene flow from adjacent sites (a trend that is particularly evident from the high divergence and limited admixture of BJA). As such, gene flow from deep water sites in Breiðafjörður may be primarily dictated by patterns of vertical migration (as seen in the migration pattern analyses), while coastal sites experience more complex gene flow patterns resulting from more dynamic hydrological conditions. Ultimately, the impact of hydrological conditions on benthic communities in Breiðafjörður has not been quantified, and therefore, more targeted studies are required to determine and quantify factors driving the migration of *B. undatum* in Breiðafjörður. Still, based on this study, population genetic structuring in Breiðafjördur is broadly stratified into two distinct population clusters corresponding to the bay's outermost and innermost geographical regions, with both clusters displaying independent signatures of inshore migration and increased migration and gene flow in the shallower, more coastal sites.

Magnúsdóttir et al. (2018) described a trend of thinner, rounder shells with relatively shorter spires, as well as increasing color diversity (and striping) from the innermost to outmost regions of Breiðafjörður. Our study showed that both color frequency distribution and shell shape distance were strongly correlated with putative population cluster (*K*) assignments. Divergence in phenotypic shell traits between putative populations can reflect random fluctuations (i.e., genetic drift) in separate populations with limited demographic connectivity, which may be further augmented by selection due to different environmental settings. Previous genetic data are congruent with a model of recent population expansion into Breiðafjörður following the last glacial maximum (Pálsson et al., 2014), where the ice sheet is considered to have extended 130 km west of the coast, as witnessed by findings of glacial moraines (Norddahl et al., 2008). Given the species' limited dispersal capabilities, the formation of multiple genetically isolated populations diverging under a genetic drift model is possible. Similar concepts have been proposed to explain the low *F*_ST_ values recorded for the ENA lineage, whereby population bottlenecks would have facilitated genetic drift, leading to low but significant population differentiation (Weetman et al., 2006). However, the inner regions of Breiðafjörður, which are typically shallower, also contain a variety of predatory crab species such as the Spider crab (*Hyas araneus*), Green crab (*Carcinus maenas*), and Atlantic Rock crab (*Cancer irroratus*). Thicker, more elongate shells with smaller apertures were hypothesized to occur in association with the predatory crab species in the innermost regions of Breiðafjörður (Bourdeau & Johansson, 2012; Magnúsdóttir et al., 2018; Thomas & Himmelman, 1988). At greater depths, negative selection by visual predators may be less intense, allowing for greater shell color diversity to be represented by individuals inhabiting the outermost regions of Breiðafjörður. In the presence of visual predators, negative selection may also constrain the frequency of particular color morphs within Breiðafjörður while selecting for specific shell shapes. Future studies should seek to characterize the relationship between genetic variation and shell shape and color in Breiðafjörður using genome‐wide association studies. In particular, GWAS studies should seek to link genetic variation with specific color morphs in a bid to characterize the distribution and frequency of color‐linked genes across Breiðafjörður.

The current study undertook the first genome‐wide analysis of population structure and divergence in *B. undatum*. Our genetic works reinforce the need to re‐examine *B. undatum* as a singular trans North Atlantic species, with Canadian and ENA whelk likely representing a potentially cryptic species complex. A clear pattern of fine‐scale population genetic structuring following a model of isolation‐by‐distance was established as a persistent trend across both small‐ (Breiðafjörður) and large‐scale (North Atlantic) geographical distances. Clear population genetic structure was defined for *B. undatum* within Breiðafjörður, Iceland, constituting two distinct putative genetic clusters. The frequency distribution of shell color and shell shape distance was significantly correlated with the two putative genetic clusters in Breiðafjörður. The work detailed here provides a rigid framework for ongoing associative studies of genetic variation in the subtidal mollusk, *B. undatum*, while laying the foundations for future comparative studies aimed at characterizing genotype–phenotype associations in common whelk.

## CONFLICT OF INTEREST

None to declare.

## AUTHOR CONTRIBUTION

**Jake Goodall:** Conceptualization (equal); Data curation (equal); Formal analysis (lead); Methodology (equal); Project administration (equal); Validation (lead); Visualization (lead); Writing‐original draft (lead); Writing‐review & editing (lead). **Kristen M. Westfall:** Conceptualization (equal); Data curation (equal); Formal analysis (supporting); Funding acquisition (supporting); Investigation (equal); Methodology (equal); Project administration (equal); Validation (supporting); Visualization (supporting); Writing‐original draft (supporting); Writing‐review & editing (supporting). **Hildur Magnúsdóttir:** Conceptualization (equal); Data curation (equal); Formal analysis (supporting); Funding acquisition (supporting); Investigation (equal); Methodology (equal); Project administration (equal); Writing‐original draft (supporting); Writing‐review & editing (supporting). **Snæbjörn Pálsson:** Conceptualization (equal); Formal analysis (supporting); Funding acquisition (supporting); Methodology (equal); Project administration (equal); Resources (supporting); Supervision (equal); Writing‐original draft (supporting); Writing‐review & editing (supporting). **Erla Björk Örnólfsdóttir:** Conceptualization (equal); Funding acquisition (lead); Project administration (equal); Resources (lead); Supervision (equal); Writing‐original draft (supporting); Writing‐review & editing (supporting). **Zophonías O. Jónsson:** Conceptualization (equal); Data curation (supporting); Formal analysis (supporting); Funding acquisition (supporting); Investigation (equal); Methodology (equal); Project administration (equal); Resources (supporting); Supervision (equal); Writing‐original draft (supporting); Writing‐review & editing (supporting).

## Supporting information

Supinfo

## Data Availability

Demultiplexed Illumina sequences are available from the European Nucleotide Archive (ENA) via study accession: PRJEB37370 (ERP120679).
