# Supplementary material for: RAD sequencing of common whelk, Buccinum undatum, reveals fine‐scale population structuring in Europe and cryptic speciation within the North Atlantic
Source: Ecol Evol. 2021 Feb 9;11(6):2616–29. doi: 10.1002/ece3.7219 (PMC7981227; doi:10.1002/ece3.7219)
Supplement: Supplementary file 1 — Supinfo [file ECE3-11-2616-s001.docx]

# SUPPLEMENTARY TABLES

**Supplementary Figure 1(A).** Summary of individual sample (n=383) read counts post demultiplexing, and usage status following quality filtering.

| **Sample** | **POP** | **Site** | **Read Count** | **Present in Primary Analysis** | **QC Filtering Stage** |
| --- | --- | --- | --- | --- | --- |
| CAN-BP3 | CAN | - | 1629655 | YES | - |
| CAN-BP5 | CAN | - | 2561875 | YES | - |
| CAN-GG3 | CAN | - | 2611268 | YES | - |
| CAN-GG4 | CAN | - | 3064500 | YES | - |
| CAN-BP2 | CAN | - | 3188581 | YES | - |
| CAN-PB4 | CAN | - | 3465011 | YES | - |
| CAN-GG1 | CAN | - | 3642037 | YES | - |
| CAN-BP6 | CAN | - | 3668265 | YES | - |
| CAN-GB1 | CAN | - | 3765041 | YES | - |
| CAN-PB2 | CAN | - | 4019133 | YES | - |
| CAN-GB3 | CAN | - | 4065655 | YES | - |
| CAN-BP4 | CAN | - | 4485466 | YES | - |
| CAN-P2 | CAN | - | 4549600 | YES | - |
| CAN-P5 | CAN | - | 4552150 | YES | - |
| CAN-GB4 | CAN | - | 4715651 | YES | - |
| CAN-P1 | CAN | - | 4814214 | YES | - |
| CAN-P6 | CAN | - | 5015972 | YES | - |
| CAN-GB2 | CAN | - | 5067569 | YES | - |
| CAN-PB5 | CAN | - | 5132650 | YES | - |
| CAN-GG2 | CAN | - | 5512511 | YES | - |
| CAN-PB3 | CAN | - | 5701721 | YES | - |
| CAN-BP1 | CAN | - | 5800697 | YES | - |
| CAN-P4 | CAN | - | 5907328 | YES | - |
| CAN-PB1 | CAN | - | 6080110 | YES | - |
| CAN-P3 | CAN | - | 6466606 | YES | - |
| CAN-GB6 | CAN | - | 6497186 | YES | - |
| CAN-GB5 | CAN | - | 6531334 | YES | - |
| CAN-PB6 | CAN | - | 6652118 | YES | - |
| CAN-GG5 | CAN | - | 2153218 | NO | Initial Pass |
| CAN-GG6 | CAN | - | 2500714 | NO | Initial Pass |
| ENG-6026 | ENG | - | 3836892 | YES | - |
| ENG-6032 | ENG | - | 4198594 | YES | - |
| ENG-6048 | ENG | - | 4408118 | YES | - |
| ENG-6049 | ENG | - | 4796624 | YES | - |
| ENG-6025 | ENG | - | 5324436 | YES | - |
| ENG-6031 | ENG | - | 5621805 | YES | - |
| ENG-6046 | ENG | - | 5935370 | YES | - |
| ENG-6044 | ENG | - | 6060076 | YES | - |
| ENG-6022 | ENG | - | 6161217 | YES | - |
| ENG-6047 | ENG | - | 6616149 | YES | - |
| ENG-6020 | ENG | - | 7598198 | YES | - |
| ENG-6023 | ENG | - | 8278549 | YES | - |
| ENG-6040 | ENG | - | 9502459 | YES | - |
| ENG-6041 | ENG | - | 13333059 | YES | - |
| ENG-6029 | ENG | - | 1752371 | NO | Initial Pass |
| ENG-6039 | ENG | - | 2660432 | NO | Initial Pass |
| ENG-6037 | ENG | - | 2978026 | NO | Initial Pass |
| ENG-6027 | ENG | - | 3175646 | NO | Initial Pass |
| ENG-6038 | ENG | - | 3181151 | NO | Initial Pass |
| ENG-6034 | ENG | - | 3354202 | NO | Initial Pass |

**Supplementary Figure 1(B).** Summary of individual sample (n=383) read counts post demultiplexing, and usage status following quality filtering.

| **Sample** | **POP** | **Site** | **Read Count** | **Present in Primary Analysis** | **QC Filtering Stage** |
| --- | --- | --- | --- | --- | --- |
| ENG-6036 | ENG | - | 3656233 | NO | Initial Pass |
| ENG-6021 | ENG | - | 4405703 | NO | Initial Pass |
| ENG-6035 | ENG | - | 5037342 | NO | Initial Pass |
| ENG-6024 | ENG | - | 5405318 | NO | Initial Pass |
| ENG-6028 | ENG | - | 6065549 | NO | Initial Pass |
| ENG-6045 | ENG | - | 6713657 | NO | Initial Pass |
| ENG-6043 | ENG | - | 6750182 | NO | Initial Pass |
| ENG-6033 | ENG | - | 6895082 | NO | Initial Pass |
| ENG-6030 | ENG | - | 7323265 | NO | Initial Pass |
| ENG-6042 | ENG | - | 12939809 | NO | Initial Pass |
| FI-7 | FAR | - | 3393082 | YES | - |
| FI-6 | FAR | - | 3445185 | YES | - |
| FI-29 | FAR | - | 3569209 | YES | - |
| FI-14 | FAR | - | 3931287 | YES | - |
| FI-23 | FAR | - | 4127366 | YES | - |
| FI-18 | FAR | - | 4156791 | YES | - |
| FI-20 | FAR | - | 4636471 | YES | - |
| FI-30 | FAR | - | 5126185 | YES | - |
| FI-9 | FAR | - | 5159587 | YES | - |
| FI-12 | FAR | - | 5431612 | YES | - |
| FI-3 | FAR | - | 5520461 | YES | - |
| FI-24 | FAR | - | 6340907 | YES | - |
| FI-1 | FAR | - | 7056018 | YES | - |
| FI-17 | FAR | - | 8483902 | YES | - |
| FI-13 | FAR | - | 9993071 | YES | - |
| FI-4 | FAR | - | 10386840 | YES | - |
| FI-15 | FAR | - | 11902055 | YES | - |
| FI-22 | FAR | - | 11930612 | YES | - |
| FI-28 | FAR | - | 1438481 | NO | Initial Pass |
| FI-2 | FAR | - | 2726739 | NO | Initial Pass |
| FI-8 | FAR | - | 3027804 | NO | Initial Pass |
| FI-11 | FAR | - | 3550479 | NO | Initial Pass |
| FI-19 | FAR | - | 4676700 | NO | Initial Pass |
| FI-5 | FAR | - | 4964269 | NO | Initial Pass |
| FI-25 | FAR | - | 5168854 | NO | Initial Pass |
| FI-21 | FAR | - | 5258966 | NO | Initial Pass |
| FI-16 | FAR | - | 5777494 | NO | Initial Pass |
| FI-31 | FAR | - | 9507792 | NO | Initial Pass |
| IS-Bja617 | ICE | BJA | 1666858 | YES | - |
| IS-Bja4 | ICE | BJA | 1752453 | YES | - |
| IS-Bja619 | ICE | BJA | 1849299 | YES | - |
| IS-Bja2 | ICE | BJA | 2011139 | YES | - |
| IS-Bja3 | ICE | BJA | 2013224 | YES | - |
| IS-Bja1 | ICE | BJA | 2553247 | YES | - |
| IS-Bja9 | ICE | BJA | 2801056 | YES | - |
| IS-Bja10 | ICE | BJA | 3173851 | YES | - |
| IS-Bja1213 | ICE | BJA | 13621807 | YES | - |
| IS-Bja608 | ICE | BJA | 112493 | NO | Initial Pass |
| IS-Bja611 | ICE | BJA | 165484 | NO | Initial Pass |
| IS-Bja621 | ICE | BJA | 179085 | NO | Initial Pass |
| IS-Bja614 | ICE | BJA | 195079 | NO | Initial Pass |
| IS-Bja618 | ICE | BJA | 222771 | NO | Initial Pass |

**Supplementary Figure 1(C).** Summary of individual sample (n=383) read counts post demultiplexing, and usage status following quality filtering.

| **Sample** | **POP** | **Site** | **Read Count** | **Present in Primary Analysis** | **QC Filtering Stage** |
| --- | --- | --- | --- | --- | --- |
| IS-Bja613 | ICE | BJA | 231708 | NO | Initial Pass |
| IS-Bja609 | ICE | BJA | 358124 | NO | Initial Pass |
| IS-Bja606 | ICE | BJA | 378569 | NO | Initial Pass |
| IS-Bja605 | ICE | BJA | 397728 | NO | Initial Pass |
| IS-Bja620 | ICE | BJA | 449343 | NO | Initial Pass |
| IS-Bja615 | ICE | BJA | 525164 | NO | Initial Pass |
| IS-Bja607 | ICE | BJA | 564215 | NO | Initial Pass |
| IS-Bja612 | ICE | BJA | 593085 | NO | Initial Pass |
| IS-Bja616 | ICE | BJA | 745639 | NO | Initial Pass |
| IS-Bja622 | ICE | BJA | 756280 | NO | Initial Pass |
| IS-Bja6 | ICE | BJA | 835800 | NO | Primary Analysis |
| IS-Bja5 | ICE | BJA | 864303 | NO | Primary Analysis |
| IS-Bja610 | ICE | BJA | 903900 | NO | Initial Pass |
| IS-Bja8 | ICE | BJA | 1063493 | NO | Initial Pass |
| IS-Bja7 | ICE | BJA | 1071728 | NO | Initial Pass |
| IS-Bja11 | ICE | BJA | 2177075 | NO | Initial Pass |
| IS-Bja1212 | ICE | BJA | 8688627 | NO | Initial Pass |
| IS-Brj713 | ICE | BRJ | 1751601 | YES | - |
| IS-Brj11 | ICE | BRJ | 1973380 | YES | - |
| IS-Brj14 | ICE | BRJ | 2782996 | YES | - |
| IS-Brj1 | ICE | BRJ | 3350442 | YES | - |
| IS-Brj13 | ICE | BRJ | 3997355 | YES | - |
| IS-Brj10 | ICE | BRJ | 4280602 | YES | - |
| IS-Brj729 | ICE | BRJ | 4406909 | YES | - |
| IS-Brj5 | ICE | BRJ | 5496963 | YES | - |
| IS-Brj16 | ICE | BRJ | 10614684 | YES | - |
| IS-Brj656 | ICE | BRJ | 8036 | NO | Initial Pass |
| IS-Brj952 | ICE | BRJ | 18602 | NO | Initial Pass |
| IS-Brj954 | ICE | BRJ | 33393 | NO | Initial Pass |
| IS-Brj683 | ICE | BRJ | 49026 | NO | Initial Pass |
| IS-Brj745 | ICE | BRJ | 57329 | NO | Initial Pass |
| IS-Brj940 | ICE | BRJ | 66296 | NO | Initial Pass |
| IS-Brj935 | ICE | BRJ | 88265 | NO | Initial Pass |
| IS-Brj674 | ICE | BRJ | 105155 | NO | Initial Pass |
| IS-Brj753 | ICE | BRJ | 111000 | NO | Initial Pass |
| IS-Brj765 | ICE | BRJ | 129311 | NO | Initial Pass |
| IS-Brj721 | ICE | BRJ | 130775 | NO | Initial Pass |
| IS-Brj682 | ICE | BRJ | 134535 | NO | Initial Pass |
| IS-Brj943 | ICE | BRJ | 141467 | NO | Initial Pass |
| IS-Brj941 | ICE | BRJ | 143405 | NO | Initial Pass |
| IS-Brj723 | ICE | BRJ | 183285 | NO | Initial Pass |
| IS-Brj734 | ICE | BRJ | 195407 | NO | Initial Pass |
| IS-Brj937 | ICE | BRJ | 195816 | NO | Initial Pass |
| IS-Brj660 | ICE | BRJ | 223706 | NO | Initial Pass |
| IS-Brj737 | ICE | BRJ | 263611 | NO | Initial Pass |
| IS-Brj711 | ICE | BRJ | 265593 | NO | Initial Pass |
| IS-Brj712 | ICE | BRJ | 268139 | NO | Initial Pass |
| IS-Brj944 | ICE | BRJ | 285538 | NO | Initial Pass |
| IS-Brj962 | ICE | BRJ | 328831 | NO | Initial Pass |
| IS-Brj725 | ICE | BRJ | 369622 | NO | Initial Pass |
| IS-Brj670 | ICE | BRJ | 424708 | NO | Initial Pass |
| IS-Brj722 | ICE | BRJ | 448559 | NO | Initial Pass |

**Supplementary Figure 1(D).** Summary of individual sample (n=383) read counts post demultiplexing, and usage status following quality filtering.

| **Sample** | **POP** | **Site** | **Read Count** | **Present in Primary Analysis** | **QC Filtering Stage** |
| --- | --- | --- | --- | --- | --- |
| IS-Brj640 | ICE | BRJ | 450845 | NO | Initial Pass |
| IS-Brj963 | ICE | BRJ | 461330 | NO | Initial Pass |
| IS-Brj969 | ICE | BRJ | 480480 | NO | Initial Pass |
| IS-Brj700 | ICE | BRJ | 558887 | NO | Initial Pass |
| IS-Brj643 | ICE | BRJ | 571298 | NO | Initial Pass |
| IS-Brj686 | ICE | BRJ | 707271 | NO | Initial Pass |
| IS-Brj8 | ICE | BRJ | 749954 | NO | Initial Pass |
| IS-Brj977 | ICE | BRJ | 913988 | NO | Initial Pass |
| IS-Brj761 | ICE | BRJ | 931604 | NO | Initial Pass |
| IS-Brj624 | ICE | BRJ | 997974 | NO | Initial Pass |
| IS-Brj938 | ICE | BRJ | 1201336 | NO | Initial Pass |
| IS-Brj7 | ICE | BRJ | 1351438 | NO | Initial Pass |
| IS-Brj3 | ICE | BRJ | 1533138 | NO | Initial Pass |
| IS-Brj9 | ICE | BRJ | 1869225 | NO | Primary Analysis |
| IS-Brj705 | ICE | BRJ | 1960528 | NO | Initial Pass |
| IS-Brj12 | ICE | BRJ | 2124187 | NO | Initial Pass |
| IS-Brj687 | ICE | BRJ | 2223528 | NO | Initial Pass |
| IS-Brj691 | ICE | BRJ | 2402988 | NO | Initial Pass |
| IS-Brj2 | ICE | BRJ | 2438939 | NO | Initial Pass |
| IS-Brj688 | ICE | BRJ | 2502804 | NO | Initial Pass |
| IS-Brj6 | ICE | BRJ | 2961621 | NO | Initial Pass |
| IS-Brj15 | ICE | BRJ | 2965464 | NO | Initial Pass |
| IS-Brj710 | ICE | BRJ | 2979546 | NO | Initial Pass |
| IS-Brj719 | ICE | BRJ | 3304113 | NO | Initial Pass |
| IS-Brj4 | ICE | BRJ | 5148063 | NO | Initial Pass |
| IS-Hru14 | ICE | HRU | 3069575 | YES | - |
| IS-Hru30 | ICE | HRU | 3147752 | YES | - |
| IS-Hru4 | ICE | HRU | 3165502 | YES | - |
| IS-Hru10 | ICE | HRU | 3284279 | YES | - |
| IS-Hru29 | ICE | HRU | 3397084 | YES | - |
| IS-Hru19 | ICE | HRU | 3429924 | YES | - |
| IS-Hru12 | ICE | HRU | 3578924 | YES | - |
| IS-Hru17 | ICE | HRU | 3876872 | YES | - |
| IS-Hru18 | ICE | HRU | 3917451 | YES | - |
| IS-Hru1109 | ICE | HRU | 4055229 | YES | - |
| IS-Hru15 | ICE | HRU | 4196068 | YES | - |
| IS-Hru28 | ICE | HRU | 4955196 | YES | - |
| IS-Hru21 | ICE | HRU | 5384662 | YES | - |
| IS-Hru6 | ICE | HRU | 6039520 | YES | - |
| IS-Hru26 | ICE | HRU | 6080363 | YES | - |
| IS-Hru11 | ICE | HRU | 7728140 | YES | - |
| IS-Hru23 | ICE | HRU | 10157452 | YES | - |
| IS-Hru24 | ICE | HRU | 22915450 | YES | - |
| IS-Hru1110 | ICE | HRU | 28720 | NO | Initial Pass |
| IS-Hru1157 | ICE | HRU | 91022 | NO | Initial Pass |
| IS-Hru1092 | ICE | HRU | 97204 | NO | Initial Pass |
| IS-Hru1080 | ICE | HRU | 133569 | NO | Initial Pass |
| IS-Hru1146 | ICE | HRU | 145244 | NO | Primary Analysis |
| IS-Hru1175 | ICE | HRU | 158620 | NO | Initial Pass |
| IS-Hru1084 | ICE | HRU | 191309 | NO | Initial Pass |
| IS-Hru985 | ICE | HRU | 194940 | NO | Initial Pass |
| IS-Hru1101 | ICE | HRU | 196853 | NO | Initial Pass |

**Supplementary Figure 1(E).** Summary of individual sample (n=383) read counts post demultiplexing, and usage status following quality filtering.

| **Sample** | **POP** | **Site** | **Read Count** | **Present in Primary Analysis** | **QC Filtering Stage** |
| --- | --- | --- | --- | --- | --- |
| IS-Hru1186 | ICE | HRU | 200631 | NO | Initial Pass |
| IS-Hru1118 | ICE | HRU | 249914 | NO | Initial Pass |
| IS-Hru1113 | ICE | HRU | 280648 | NO | Initial Pass |
| IS-Hru981 | ICE | HRU | 288190 | NO | Initial Pass |
| IS-Hru1075 | ICE | HRU | 304353 | NO | Initial Pass |
| IS-Hru1103 | ICE | HRU | 315153 | NO | Initial Pass |
| IS-Hru1119 | ICE | HRU | 350235 | NO | Initial Pass |
| IS-Hru1077 | ICE | HRU | 372221 | NO | Initial Pass |
| IS-Hru1188 | ICE | HRU | 388315 | NO | Initial Pass |
| IS-Hru1072 | ICE | HRU | 410708 | NO | Initial Pass |
| IS-Hru986 | ICE | HRU | 423559 | NO | Initial Pass |
| IS-Hru1076 | ICE | HRU | 433727 | NO | Initial Pass |
| IS-Hru1149 | ICE | HRU | 468569 | NO | Initial Pass |
| IS-Hru1100 | ICE | HRU | 513675 | NO | Initial Pass |
| IS-Hru994 | ICE | HRU | 604034 | NO | Initial Pass |
| IS-Hru992 | ICE | HRU | 695777 | NO | Initial Pass |
| IS-Hru1169 | ICE | HRU | 701381 | NO | Primary Analysis |
| IS-Hru1177 | ICE | HRU | 731350 | NO | Initial Pass |
| IS-Hru993A | ICE | HRU | 785328 | NO | Initial Pass |
| IS-Hru1074 | ICE | HRU | 825715 | NO | Primary Analysis |
| IS-Hru982 | ICE | HRU | 985927 | NO | Primary Analysis |
| IS-Hru16 | ICE | HRU | 1063732 | NO | Initial Pass |
| IS-Hru1071 | ICE | HRU | 1263040 | NO | Initial Pass |
| IS-Hru980 | ICE | HRU | 1267532 | NO | Initial Pass |
| IS-Hru5 | ICE | HRU | 1454256 | NO | Initial Pass |
| IS-Hru9 | ICE | HRU | 1611912 | NO | Initial Pass |
| IS-Hru987 | ICE | HRU | 2601396 | NO | Initial Pass |
| IS-Hru988 | ICE | HRU | 2902606 | NO | Initial Pass |
| IS-Hru8 | ICE | HRU | 3058631 | NO | Initial Pass |
| IS-Hru13 | ICE | HRU | 3567371 | NO | Initial Pass |
| IS-Hru20 | ICE | HRU | 3746371 | NO | Initial Pass |
| IS-Hru1 | ICE | HRU | 4099537 | NO | Initial Pass |
| IS-Hru991 | ICE | HRU | 4103876 | NO | Initial Pass |
| IS-Hru7 | ICE | HRU | 4242082 | NO | Initial Pass |
| IS-Hru25 | ICE | HRU | 4609458 | NO | Initial Pass |
| IS-Hru2 | ICE | HRU | 6519685 | NO | Initial Pass |
| IS-Hru22 | ICE | HRU | 7560431 | NO | Initial Pass |
| IS-Hru27 | ICE | HRU | 7743420 | NO | Initial Pass |
| IS-Hru3 | ICE | HRU | 8569869 | NO | Initial Pass |
| IS-Hva1302 | ICE | HVA | 1323339 | YES | - |
| IS-Hva1228 | ICE | HVA | 1327380 | YES | - |
| IS-Hva2 | ICE | HVA | 1578725 | YES | - |
| IS-Hva1246 | ICE | HVA | 1920053 | YES | - |
| IS-Hva1316 | ICE | HVA | 2022623 | YES | - |
| IS-Hva15 | ICE | HVA | 2053217 | YES | - |
| IS-Hva7 | ICE | HVA | 2056244 | YES | - |
| IS-Hva1251 | ICE | HVA | 2166992 | YES | - |
| IS-Hva1277 | ICE | HVA | 2554944 | YES | - |
| IS-Hva14 | ICE | HVA | 2590858 | YES | - |
| IS-Hva1290 | ICE | HVA | 2723518 | YES | - |
| IS-Hva1319 | ICE | HVA | 3132868 | YES | - |
| IS-Hva19 | ICE | HVA | 3491553 | YES | - |

**Supplementary Figure 1(F).** Summary of individual sample (n=383) read counts post demultiplexing, and usage status following quality filtering.

| **Sample** | **POP** | **Site** | **Read Count** | **Present in Primary Analysis** | **QC Filtering Stage** |
| --- | --- | --- | --- | --- | --- |
| IS-Hva1245 | ICE | HVA | 3810820 | YES | - |
| IS-Hva1237 | ICE | HVA | 3830135 | YES | - |
| IS-Hva1278 | ICE | HVA | 3915446 | YES | - |
| IS-Hva17 | ICE | HVA | 3949135 | YES | - |
| IS-Hva1254 | ICE | HVA | 3959042 | YES | - |
| IS-Hva24 | ICE | HVA | 3979586 | YES | - |
| IS-Hva1223 | ICE | HVA | 4204191 | YES | - |
| IS-Hva1308 | ICE | HVA | 4348074 | YES | - |
| IS-Hva1235 | ICE | HVA | 4726700 | YES | - |
| IS-Hva16 | ICE | HVA | 4765607 | YES | - |
| IS-Hva6 | ICE | HVA | 4866609 | YES | - |
| IS-Hva1226 | ICE | HVA | 5645406 | YES | - |
| IS-Hva26 | ICE | HVA | 5686654 | YES | - |
| IS-Hva20 | ICE | HVA | 6394471 | YES | - |
| IS-Hva1238 | ICE | HVA | 6529902 | YES | - |
| IS-Hva23 | ICE | HVA | 6974028 | YES | - |
| IS-Hva1252 | ICE | HVA | 7046002 | YES | - |
| IS-Hva4 | ICE | HVA | 7459087 | YES | - |
| IS-Hva1 | ICE | HVA | 7560369 | YES | - |
| IS-Hva21 | ICE | HVA | 7916046 | YES | - |
| IS-Hva9 | ICE | HVA | 8687852 | YES | - |
| IS-Hva10 | ICE | HVA | 769716 | NO | Initial Pass |
| IS-Hva1243 | ICE | HVA | 868638 | NO | Primary Analysis |
| IS-Hva22 | ICE | HVA | 980776 | NO | Primary Analysis |
| IS-Hva1272 | ICE | HVA | 1294438 | NO | Primary Analysis |
| IS-Hva1265 | ICE | HVA | 1647766 | NO | Initial Pass |
| IS-Hva8 | ICE | HVA | 1885541 | NO | Initial Pass |
| IS-Hva1264 | ICE | HVA | 1891228 | NO | Initial Pass |
| IS-Hva18 | ICE | HVA | 2453931 | NO | Initial Pass |
| IS-Hva1294 | ICE | HVA | 2743273 | NO | Initial Pass |
| IS-Hva1250 | ICE | HVA | 2938308 | NO | Initial Pass |
| IS-Hva3 | ICE | HVA | 3358579 | NO | Initial Pass |
| IS-Hva27 | ICE | HVA | 3442247 | NO | Initial Pass |
| IS-Hva1236 | ICE | HVA | 3604215 | NO | Initial Pass |
| IS-Hva1247 | ICE | HVA | 3651857 | NO | Initial Pass |
| IS-Hva1293 | ICE | HVA | 4290906 | NO | Initial Pass |
| IS-Hva12 | ICE | HVA | 4764673 | NO | Initial Pass |
| IS-Hva1262 | ICE | HVA | 4860103 | NO | Initial Pass |
| IS-Hva13 | ICE | HVA | 5520109 | NO | Initial Pass |
| IS-Hva1263 | ICE | HVA | 5658258 | NO | Initial Pass |
| IS-Hva5 | ICE | HVA | 5662073 | NO | Initial Pass |
| IS-Hva25 | ICE | HVA | 7058631 | NO | Initial Pass |
| IS-Hva1283 | ICE | HVA | 7397608 | NO | Initial Pass |
| IS-Hva11 | ICE | HVA | 9212997 | NO | Initial Pass |
| IS-Hva1273 | ICE | HVA | 14010387 | NO | Initial Pass |
| IS-Odd860 | ICE | ODD | 2462146 | YES | - |
| IS-Odd2 | ICE | ODD | 5078138 | YES | - |
| IS-Odd3 | ICE | ODD | 5411449 | YES | - |
| IS-Odd7 | ICE | ODD | 9591503 | YES | - |
| IS-Odd5 | ICE | ODD | 76602 | NO | Initial Pass |
| IS-Odd797 | ICE | ODD | 150576 | NO | Initial Pass |
| IS-Odd832 | ICE | ODD | 170963 | NO | Initial Pass |

**Supplementary Figure 1(G).** Summary of individual sample (n=383) read counts post demultiplexing, and usage status following quality filtering.

| **Sample** | **POP** | **Site** | **Read Count** | **Present in Primary Analysis** | **QC Filtering Stage** |
| --- | --- | --- | --- | --- | --- |
| IS-Odd838 | ICE | ODD | 180809 | NO | Initial Pass |
| IS-Odd918 | ICE | ODD | 197014 | NO | Initial Pass |
| IS-Odd871 | ICE | ODD | 243422 | NO | Initial Pass |
| IS-Odd808 | ICE | ODD | 256062 | NO | Initial Pass |
| IS-Odd777 | ICE | ODD | 282376 | NO | Initial Pass |
| IS-Odd847 | ICE | ODD | 429201 | NO | Initial Pass |
| IS-Odd850 | ICE | ODD | 438337 | NO | Initial Pass |
| IS-Odd801 | ICE | ODD | 449252 | NO | Initial Pass |
| IS-Odd842 | ICE | ODD | 467457 | NO | Initial Pass |
| IS-Odd779 | ICE | ODD | 482506 | NO | Initial Pass |
| IS-Odd824 | ICE | ODD | 500259 | NO | Initial Pass |
| IS-Odd878 | ICE | ODD | 530206 | NO | Initial Pass |
| IS-Odd802 | ICE | ODD | 610961 | NO | Initial Pass |
| IS-Odd876 | ICE | ODD | 677806 | NO | Primary Analysis |
| IS-Odd849 | ICE | ODD | 682924 | NO | Initial Pass |
| IS-Odd811 | ICE | ODD | 744875 | NO | Initial Pass |
| IS-Odd815 | ICE | ODD | 781401 | NO | Initial Pass |
| IS-Odd800 | ICE | ODD | 815106 | NO | Initial Pass |
| IS-Odd821 | ICE | ODD | 881131 | NO | Initial Pass |
| IS-Odd818 | ICE | ODD | 891262 | NO | Initial Pass |
| IS-Odd813 | ICE | ODD | 913722 | NO | Initial Pass |
| IS-Odd770 | ICE | ODD | 1042506 | NO | Initial Pass |
| IS-Odd868 | ICE | ODD | 1057340 | NO | Initial Pass |
| IS-Odd822 | ICE | ODD | 1094816 | NO | Initial Pass |
| IS-Odd789 | ICE | ODD | 1144609 | NO | Initial Pass |
| IS-Odd839 | ICE | ODD | 1163530 | NO | Initial Pass |
| IS-Odd828 | ICE | ODD | 1284522 | NO | Initial Pass |
| IS-Odd841 | ICE | ODD | 1685645 | NO | Initial Pass |
| IS-Odd780 | ICE | ODD | 3113780 | NO | Initial Pass |
| IS-Odd792 | ICE | ODD | 3308583 | NO | Initial Pass |
| IS-Odd4 | ICE | ODD | 4575306 | NO | Initial Pass |
| IS-Odd6 | ICE | ODD | 5228338 | NO | Initial Pass |
| IS-Odd1 | ICE | ODD | 6376228 | NO | Initial Pass |
| IS-Rau1217 | ICE | RAU | 3255565 | YES | - |
| IS-Rau1216 | ICE | RAU | 4487309 | YES | - |
| IS-Rau1220 | ICE | RAU | 6203238 | YES | - |
| IS-Rau1218 | ICE | RAU | 7031855 | YES | - |
| IS-Rau1219 | ICE | RAU | 7920527 | YES | - |
| IS-Rau1215 | ICE | RAU | 8374683 | YES | - |
| IS-Rau1221 | ICE | RAU | 7064548 | NO | Initial Pass |
| IS-Rau1214 | ICE | RAU | 8602890 | NO | Initial Pass |
| IS-Sko9 | ICE | SKO | 2031790 | YES | - |
| IS-Sko4 | ICE | SKO | 2982711 | YES | - |
| IS-Sko14 | ICE | SKO | 3252934 | YES | - |
| IS-Sko1200 | ICE | SKO | 3530908 | YES | - |
| IS-Sko1 | ICE | SKO | 3875025 | YES | - |
| IS-Sko2 | ICE | SKO | 3996217 | YES | - |
| IS-Sko5 | ICE | SKO | 4083404 | YES | - |
| IS-Sko11 | ICE | SKO | 4367790 | YES | - |
| IS-Sko3 | ICE | SKO | 4375937 | YES | - |
| IS-Sko1210 | ICE | SKO | 4536373 | YES | - |
| IS-Sko1201 | ICE | SKO | 4608616 | YES | - |

**Supplementary Figure 1(H).** Summary of individual sample (n=383) read counts post demultiplexing, and usage status following quality filtering.

| **Sample** | **POP** | **Site** | **Read Count** | **Present in Primary Analysis** | **QC Filtering Stage** |
| --- | --- | --- | --- | --- | --- |
| IS-Sko12 | ICE | SKO | 4683071 | YES | - |
| IS-Sko1204 | ICE | SKO | 5203996 | YES | - |
| IS-Sko13 | ICE | SKO | 6414485 | YES | - |
| IS-Sko1207 | ICE | SKO | 6664328 | YES | - |
| IS-Sko1208 | ICE | SKO | 6873151 | YES | - |
| IS-Sko1209 | ICE | SKO | 7037801 | YES | - |
| IS-Sko1202 | ICE | SKO | 8454566 | YES | - |
| IS-Sko1211 | ICE | SKO | 9269795 | YES | - |
| IS-Sko1196 | ICE | SKO | 9514543 | YES | - |
| IS-Sko1199 | ICE | SKO | 9746856 | YES | - |
| IS-Sko1195 | ICE | SKO | 11863525 | YES | - |
| IS-Sko1194 | ICE | SKO | 13063102 | YES | - |
| IS-Sko1198 | ICE | SKO | 13124086 | YES | - |
| IS-Sko8 | ICE | SKO | 1193605 | NO | Primary Analysis |
| IS-Sko7 | ICE | SKO | 1563503 | NO | Primary Analysis |
| IS-Sko10 | ICE | SKO | 1755041 | NO | Initial Pass |
| IS-Sko6 | ICE | SKO | 2018311 | NO | Initial Pass |
| IS-Sko1203 | ICE | SKO | 2364061 | NO | Initial Pass |
| IS-Sko1197 | ICE | SKO | 5856375 | NO | Initial Pass |
| IS-Sko1206 | ICE | SKO | 6824349 | NO | Initial Pass |
| IS-Sko1205 | ICE | SKO | 7491738 | NO | Initial Pass |

**Supplementary Table 2.** Filtering parameters and outputs for the initial *Radiator* filtering of genetic data. The initial pass was primarily focused on detecting and removing low-quality individuals from downstream analyses.

| Filter Parameter | Input/ Threshold | # Individuals Blacklisted | # Individuals After | # Locus Blacklisted | # Locus After | # SNP Blacklisted | # SNP After |
| --- | --- | --- | --- | --- | --- | --- | --- |
| Unfiltered | - | - | 383 | - | 51530 | - | 244648 |
| Monomorphic markers | TRUE | 0 | 383 | 63 | 51467 | 379 | 244269 |
| Common markers | TRUE | 0 | 383 | 29523 | 21944 | 146361 | 97908 |
| Filter individuals poorly genotyped | Yes; OutlierStatistics | 0 | 383 | 0 | 21944 | 0 | 97908 |
| Filter markers based on MAC | MIN: 7 | 0 | 383 | 5089 | 16855 | 50886 | 47022 |
| Coverage | MIN: 7; MAX:50 | 0 | 383 | 410 | 16445 | 1057 | 45965 |
| Filter Genotyping Proportion | MIN: 0.4 | 0 | 383 | 3571 | 12874 | 11327 | 34638 |
| Filter SNP position on read | Outliers | 0 | 383 | 0 | 12874 | 0 | 34638 |
| Filter max SNP per locus | Yes; Outlier Statistics | 0 | 383 | 47 | 12827 | 726 | 33912 |
| SNP per read/ Linkage disequilibrium | MAC | 0 | 383 | 0 | 12827 | 21085 | 12827 |
| Mixed Genomes | MIN: 0.07; MAX: 0.14 | 35 | 348 | 0 | 12827 | 0 | 12827 |
| Duplicate Genomes * | 0.01 | 171 | 177 | 0 | 12827 | 0 | 12827 |
| Final Output File | - | - | 177 | - | 12827 | - | 12827 |

* calculates a relative distance (manhattan) metric based on individuals' distance divided by the maximum distance observed in the dataset (not calculated by strata). Note that blacklisting can be influenced by one or more individuals per pair containing a low proportion of genotypes and a lack of shared markers.

**Supplementary Table 3.** Filtering parameters and outputs of primary *populations* genetic analyses. The primary dataset's quality filtering sought the removal of any low-quality individuals or loci remaining after the initial quality filtering pass.

| Filter Parameter | Input/ Threshold | # Individuals Blacklisted | # Individuals After | # Locus Blacklisted | # Locus After | # SNP Blacklisted | # SNP After |
| --- | --- | --- | --- | --- | --- | --- | --- |
| Unfiltered | - | - | 177 | - | 71757 | - | 346020 |
| Monomorphic markers | TRUE | 0 | 177 | 92 | 71665 | 652 | 345368 |
| Common markers | TRUE | 0 | 177 | 24347 | 47318 | 109475 | 235893 |
| Filter individuals poorly genotyped | Yes; Outlier Statistics | 13 | 164 | 62 | 47256 | 984 | 234909 |
| Filter markers based on MAC | MIN: 7 | 0 | 164 | 10070 | 37186 | 124624 | 110285 |
| Coverage | MIN: 7; MAX:40 | 0 | 164 | 1158 | 36028 | 3531 | 106754 |
| Filter SNP position on read | Outliers | 0 | 164 | 0 | 36028 | 2 | 106752 |
| Filter max SNP per locus | Yes; Outlier Statistics | 0 | 164 | 192 | 35836 | 3388 | 103364 |
| SNP per read/ Linkage disequilibrium | MAC | 0 | 164 | 0 | 35836 | 67528 | 35836 |
| Final Output File | - | - | 164 | - | 35836 | - | 35836 |

**Supplementary Table 4.** Frequency of shell color of whelk sampled in Breiðafjörður, Iceland. Populations are designated relative to their sample site of origin with sites denoted as BJA (Bjarneyjaráll), BRJ (Brjánslækur), HVA (Hvammsfjörður), HRU (Hrútey), ODD (Oddbjarnarsker), SKO (Skor). No phenotypic data could be sourced RAU (Rauðasandur), and therefore, individuals from this site were excluded from all shell color association analyses. POP 1 and 2 are representative of the putative populations assigned by the K=2 clustering.

| Shell color | BJA | BRJ | HVA | HRU | ODD | SKO | POP1 | POP2 |
| --- | --- | --- | --- | --- | --- | --- | --- | --- |
| Brown | 2 | 3 | 3 | 16 | 44 | 5 | 7 | 66 |
| Darkgrey | 1 | 0 | 0 | 0 | 2 | 0 | 1 | 2 |
| Green | 0 | 50 | 36 | 0 | 1 | 2 | 2 | 87 |
| Grey | 1 | 0 | 0 | 0 | 2 | 0 | 1 | 3 |
| Orange | 6 | 12 | 1 | 29 | 4 | 2 | 8 | 46 |
| White | 8 | 0 | 0 | 9 | 14 | 9 | 11 | 23 |
| Total | 18 | 65 | 40 | 54 | 67 | 18 | 30 | 227 |
